# Charge Transfer Kinetics in Halide Perovskites: On the Constraints of Time-Resolved Spectroscopy Measurements

**DOI:** 10.1021/acsenergylett.4c00736

**Published:** 2024-06-05

**Authors:** Xiangtian Chen, Prashant V. Kamat, Csaba Janáky, Gergely Ferenc Samu

**Affiliations:** † Department of Physical Chemistry and Materials Science, Interdisciplinary Excellence Centre, University of Szeged, Aradi Square 1, Szeged H-6720, Hungary; ‡ ELI-ALPS, ELI-HU Non-Profit Ltd., Wolfgang Sandner street 3., Szeged H-6728, Hungary; § Department of Chemistry and Biochemistry, University of Notre Dame, Notre Dame, Indiana 46556, United States; ∥ Department of Molecular and Analytical Chemistry, University of Szeged, Dóm Square 7-8. Szeged H-6721, Hungary

## Abstract

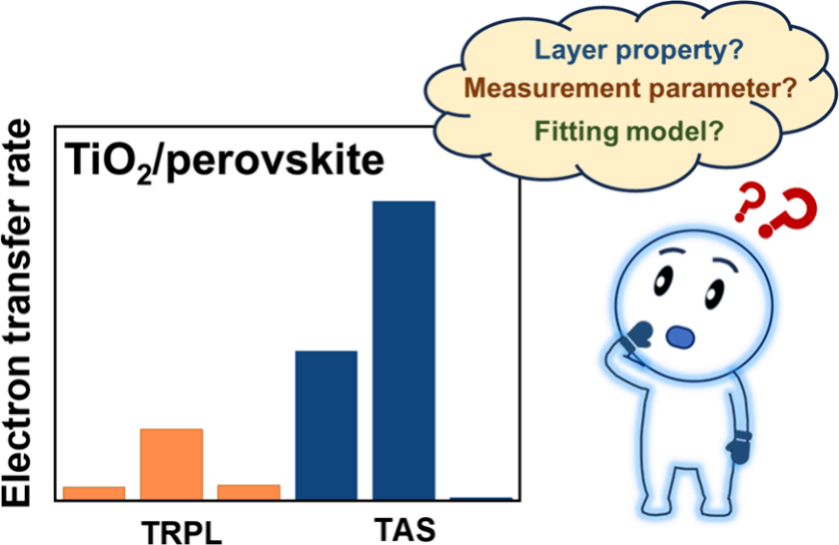

Understanding photophysical processes in lead halide perovskites is an important aspect of optimizing the performance of optoelectronic devices. The determination of exact charge carrier extraction rate constants remains elusive, as there is a large and persistent discrepancy in the reported absolute values. In this review, we concentrate on experimental procedures adopted in the literature to obtain kinetic estimates of charge transfer processes and limitations imposed by the spectroscopy technique employed. Time-resolved techniques (e.g., transient absorption–reflection and time-resolved photoluminescence spectroscopy) are commonly employed to probe charge transfer at perovskite/transport layer interfaces. The variation in sample preparation and measurement conditions can produce a wide dispersion of the measured kinetic parameters. The selected time window and the kinetic fitting model employed introduce additional uncertainty. We discuss here evaluation strategies that rely on multiexponential fitting protocols (regular or stretched) and show how the dispersion in the reported values for carrier transfer rate constants can be resolved.

As lead halide perovskites are drawing attention in solar cells, LEDs, and other optoelectronic devices, research efforts are also dedicated to understanding their photophysical properties. In such energy conversion devices, perovskite light absorbers (or emitters) are often paired with charge transport layers (CTL) that aid charge extraction (or injection) and their transport to collecting electrodes. To realize the full potential of perovskite-based devices, it is important to accurately map charge transfer across these interfaces.^1−3^ A careful balance of electron and hole transport will minimize charge accumulation and hence can maximize the photon conversion efficiency suppress degradation and ion migration^4^ in these devices.^5^ To monitor charge carrier generation following bandgap excitation and the fate of generated carriers as they are collected by the transport layers, transient spectroscopy techniques like time-resolved microwave conductivity,^6,7^ transient surface photovoltage,^8^ time-resolved photoluminescence (TRPL) and ultrafast pump–probe transient absorption/reflection (TA/TR) spectroscopy have been found to be useful. Among these methods, TRPL and ultrafast pump–probe TA/TR techniques are popular to probe the excited-state deactivation of semiconductor nanocrystals and films including charge transfer processes. Based on the sample composition and the detection methodology, the signal detection extends from picoseconds to microseconds.^9^

Photoinduced processes that dictate charge separation and charge carrier dynamics in halide perovskites are summarized in Figure 1 (together with their time scale). Metal halide perovskites (except 2D perovskites) have a small exciton binding energy; thus, excitons formed during light excitation dissociate to form free carriers at room temperature. If the excitation energy is greater than the bandgap of the semiconductor, hot carriers populate within the sample. The hot carrier population undergoes thermalization within ∼100 fs,^10^ and the thermalized carriers relax to the respective band edges of the light absorber in ∼1 ps.^11^ Carrier extraction at these early time scales remains an optimistic aspiration for developing hot-carrier devices.^12^ The cooled carriers undergo radiative charge carrier recombination or charge trapping, a process that can extend into the nanoseconds to several microseconds time scale. With a quick capture of charge carriers, efficiencies greater than the detailed balance limit could theoretically be achieved.^13^ The trapped carriers can recombine in a nonradiative step with free carriers or can participate in the photodoping of the samples.^14^ These processes can be monitored through changes in the absorption or photoluminescence by employing time-resolved spectroscopy tools.^15^ The trap-induced processes extends into the microsecond to millisecond time domain.

**Figure 1:**
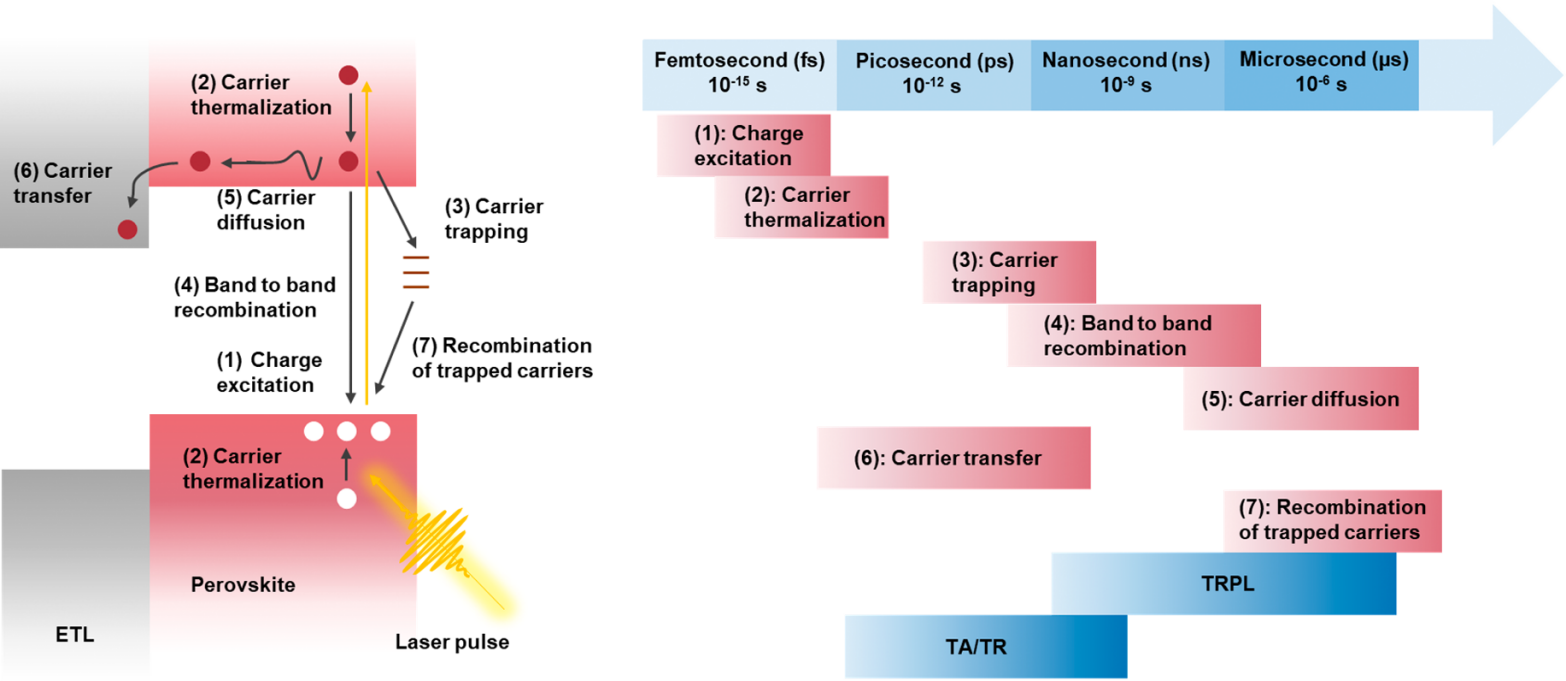
Schematic representation of the complex photophysical processes occurring in a charge transfer layer/perovskite assembly after light excitation, together with the time scale of the outlined photophysical processes (graphed together with relevant optical techniques used to study them). Our own compilation.

The population of carriers that avoid charge carrier recombination and trapping is able to diffuse or migrate to the respective CTL/perovskite interface. The driving force for this movement of charge carriers arises due to the band offsets between the perovskite absorbing layer and the charge transport layer, which imparts a built-in electrochemical bias.^16,17^ However, the charge trapping processes can compete with charge transfer, making it difficult to separate their contributions to the overall charge carrier dynamics.^18,19^ If defects are localized at the interface, they can introduce additional barriers for carrier extraction.^20^ The diffusion/migration of carriers to the extraction layer interface depends on the thickness, morphology (e.g., grain size/boundary), and preparation method of the perovskite absorber, which introduces additional uncertainty.^21^ All of these factors together manifest in a large dispersion (up to several orders of magnitude) of the carrier extraction rate constants (or related excited state lifetimes) even when the same technique is used for ostensibly the “same” material. Some selected examples are compiled in Table 1. Additional details are provided in Tables S1–S6 in the Supporting Information.

**Table 1:** Range of Reported Lifetimes (τ) Related to Charge Transfer (CT) in Different CTL/CH_3_NH_3_PbI_3_ Assemblies

method	architecture	measurement parameters	evaluation method	τ_CT_	rate constant (10^7^ s^–1^)	ref
TRPL	TiO_2_/CH_3_NH_3_PbI_3_	λ_exc_ = 625 nm, 0.1 μJ cm^–2^	CT equation was used with derived τ_effective_ (Al_2_O_3_/perovskite acted as reference without CT)	11 ns	9.1	(22)
		λ_exc_ = 464 nm, unknown fluence	triexponential fitting, the fast component is attributed to CT	2.1 ns	47.6	(23)
		λ_exc_ = 640 nm, unknown fluence	biexponential fitting, the fast component is attributed to CT	9.7 ns	10.3	(24)
						
TA	compact-TiO_2_/CH_3_NH_3_PbI_3_	λ_exc_ = 400 nm, 10 μJ cm^–2^	multiexponential fitting, the long lifetime component is attributed to CT	370 ps	270.3	(25)
		λ_exc_ = 370 nm, 3.0 μJ cm^–2^	multiexponential fitting to lower wavelength than the ground state bleach	<10 ps	>10000	(26)
		λ_exc_ = 485 nm, 0.5–75 μJ cm^–2^	global analysis of data, CT is determined from the fluence dependence of average lifetime	50 ns	2.0	(27)
						
TRPL	spiro-MeOTAD/CH_3_NH_3_PbI_3_	λ_exc_ = 600 nm, 1.3 μJ cm^–2^	CT equation was used with lifetimes determined from monoexponential fitting	0.7 ns	142.9	(28)
		λ_exc_ = 625 nm, <0.1 μJ cm^–2^	CT equation was used with derived τ_effective_ (Al_2_O_3_/perovskite acted as reference without CT)	1.8 ns	55.6	(22)
	spiro-MeOTAD/FAMA perovskite	λ_exc_ = 460 nm, 0.4 W cm^–2^	global analysis of data, CT is determined from the fluence dependence of average lifetime	100 ns	1	(29)
						
TA	spiro-MeOTAD/CH_3_NH_3_PbI_3_	λ_exc_ = 600 nm, 10.0 μJ cm^–2^	multiexponential fitting, the short lifetime component is attributed to CT	0.7 ns	142.9	(28)
		λ_exc_ = 460 nm, 2.0 μJ cm^–2^	multiexponential fitting	0.8 ps	125000	(30)
		λ_exc_ = 485 nm, 0.5–75 μJ cm^–2^	global analysis of data, CT is determined from the fluence dependence of average lifetime	17 ns	5.9	(31)

Knowledge of the precise carrier extraction rate constant is of vital importance in the design of efficient optoelectronic devices. Additionally, effects related to excitation-induced alleviation of trap states or restructuring of perovskite layers (ion accumulation, segregation) are also present in CTL/perovskite assemblies and must be considered in related studies.^32^ Furthermore, differences exist in partially assembled devices containing one extraction layer/perovskite interface and fully assembled architectures.^21^ This is mainly caused by the presence of built-in electric fields and unbalanced carrier extraction in a full assembly. Such inconsistency in measurements and kinetic analysis is less prevalent for molecular systems,^33^ yet for complex and “soft” semiconductors such as halide perovskites extra care must be taken in sample preparation and measurement.

Optical and electronic/electrochemical techniques are commonly employed to elucidate the photophysical processes in perovskite light absorbers.^34^ In this review, we examine how the kinetic parameters vary based on the chosen techniques (i.e., TA/TR spectroscopy vs TRPL spectroscopy) and the experimental conditions. We also provide ways to recognize the shortcomings of the kinetic analysis and discuss possible ways to consolidate the measurements to obtain better insights. As perovskite-based optoelectronic devices are mostly solid-state in nature, we limit the discussion to thin-film samples/devices. Notably, the large spread of carrier extraction rate constants also exists for quantum dot dispersions containing electron/hole scavenger species.^35^

## Layer Quality Matters

**Scheme 1:**
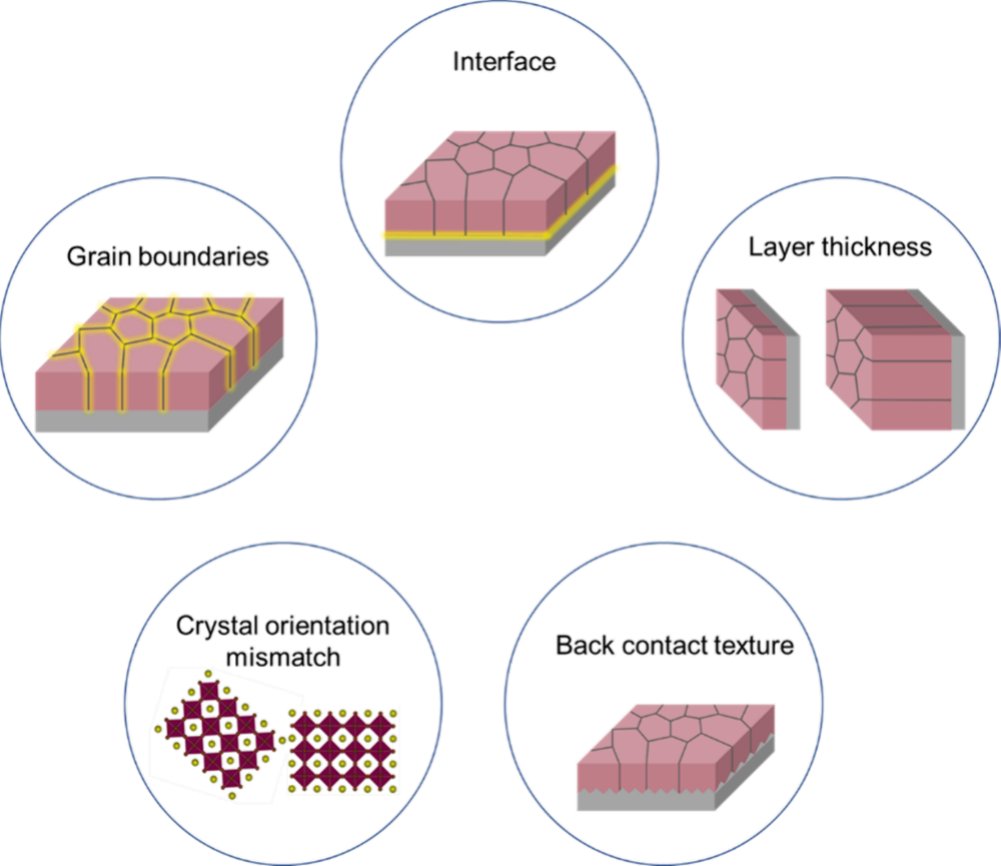
Schematic Representation of the Perovskite Layer Properties Influencing Charge Carrier Dynamics

The granular structure of the perovskite layers is rich in different interfaces, which can form various trap/defect states. These defects can arise from crystal strain (variation in orientation, packing)^36^ or element vacancies manifesting as dangling bonds.^37^ The formed trap states can participate in charge carrier recombination processes, which contribute to efficiency losses in the devices. Such processes occur on a time scale similar to that for charge extraction, which makes their separation challenging.^31^ Information about the density of these trap states of a given perovskite layer is not always immediately available from routine characterizations. Furthermore, the quantity of trap states can rarely be varied independently from other decisive layer properties (e.g., grain size, grain orientation, and interface quality), complicating the analysis of the results.^38^ It is first important to understand how the different properties of the layers can influence charge carrier dynamics.^39^ Typical properties of the perovskite layer are illustrated in Scheme 1.

The different chemical makeups of grain boundaries (element vacancies, excess of unreacted precursor complexes, dangling bonds) can result in different photophysical behaviors compared to that of the bulk. PL intensities and lifetimes were found to be lower at grain boundaries, indicating faster nonradiative decay.^40^ Also, by carefully controlling the grain size and thickness, trap-assisted charge carrier recombination was found to be more efficient in small grains since the grain boundaries became more accessible to charge carriers (e.g., on the order of the carrier diffusion length).^41^ Grain boundaries also play an important role in inducing asymmetric charge carrier transport and extraction to the collection layers. For example, laterally oriented grain boundaries can reduce hole mobility but have less impact on electron mobility, which results in asymmetries in vertical/horizontal charge transport within perovskite layers.^42^ The local orientation within a given grain can also affect the intrinsic photophysical behavior of the perovskite layers. A larger spread in crystal misorientation can result in lower PL intensity (and thus PL quantum yield), indicating that crystal mismatch sites can also act as recombination centers.^43^ At the same time, defects and trap states were found to be either neutral or in some cases beneficial for enhancing photovoltage,^44^ assisting charge transfer,^44−46^ or suppressing cation migration.^47^ Clearly grain boundaries and intrinsic defect states have an impact on the charge carrier dynamics and therefore have to be considered when reporting/comparing between different perovskite compositions and devices.

The quality of interfaces is also important, as defect states can be located at both ETL/perovskite and HTL/perovskite interfaces as well as within the perovskite film. Often charge carrier kinetic measurements are performed on partial or incomplete devices to isolate either electron or hole injection kinetics, yet the defect states located at the sample surface are often ignored. Surface-sensitive TR measurements have revealed that the total carrier lifetime in polycrystalline perovskite films is limited by charge carrier recombination at the point of excitation.^48^ By effective passivation, the nonradiative charge carrier recombination rate of polycrystalline films can be similar to or even lower than that of single-crystal perovskites.^48^ Recombination losses at interfaces can be more severe than those within the crystalline grains and at internal grain boundaries.^49,50^ To overcome these losses, efforts have been made to either employ surface chemical treatments of the perovskite absorber or insert ultrathin interlayers between the perovskite and transport layers, leading to a substantial reduction of these interfacial losses.^51^ In the case of surface chemical treatments, addition of a Lewis base helps to passivate undercoordinated Pb atoms at the surface of the perovskite through an electron-donation mechanism.^52,53^ On the other hand, ultrathin interlayers are typically composed of high-bandgap materials such as polystyrene,^54^ Ga_2_O_3_,^55^ or 2D perovskite precursors such as phenethylammonium iodide.^56^ Although the exact mechanism(s) have not been fully elucidated, it is likely that these interlayers help to passivate surface trap states, modulate extraction of majority carriers, or suppress surface recombination by impeding minority carrier injection.^51^ However, it is clear that the chemical makeup of the perovskite layer in the vicinity of the interfaces can be different than in the bulk. For example, in the case of a TiO_2_ ETL the formation of PbI_2_ impurities can often be found near the interface, influencing the charge transfer process.^57^ Thus, careful selection of interlayers and surface treatments should be considered, and such surface layer compositions and thicknesses should be taken into consideration when comparing across device compositions.

**Figure 2:**
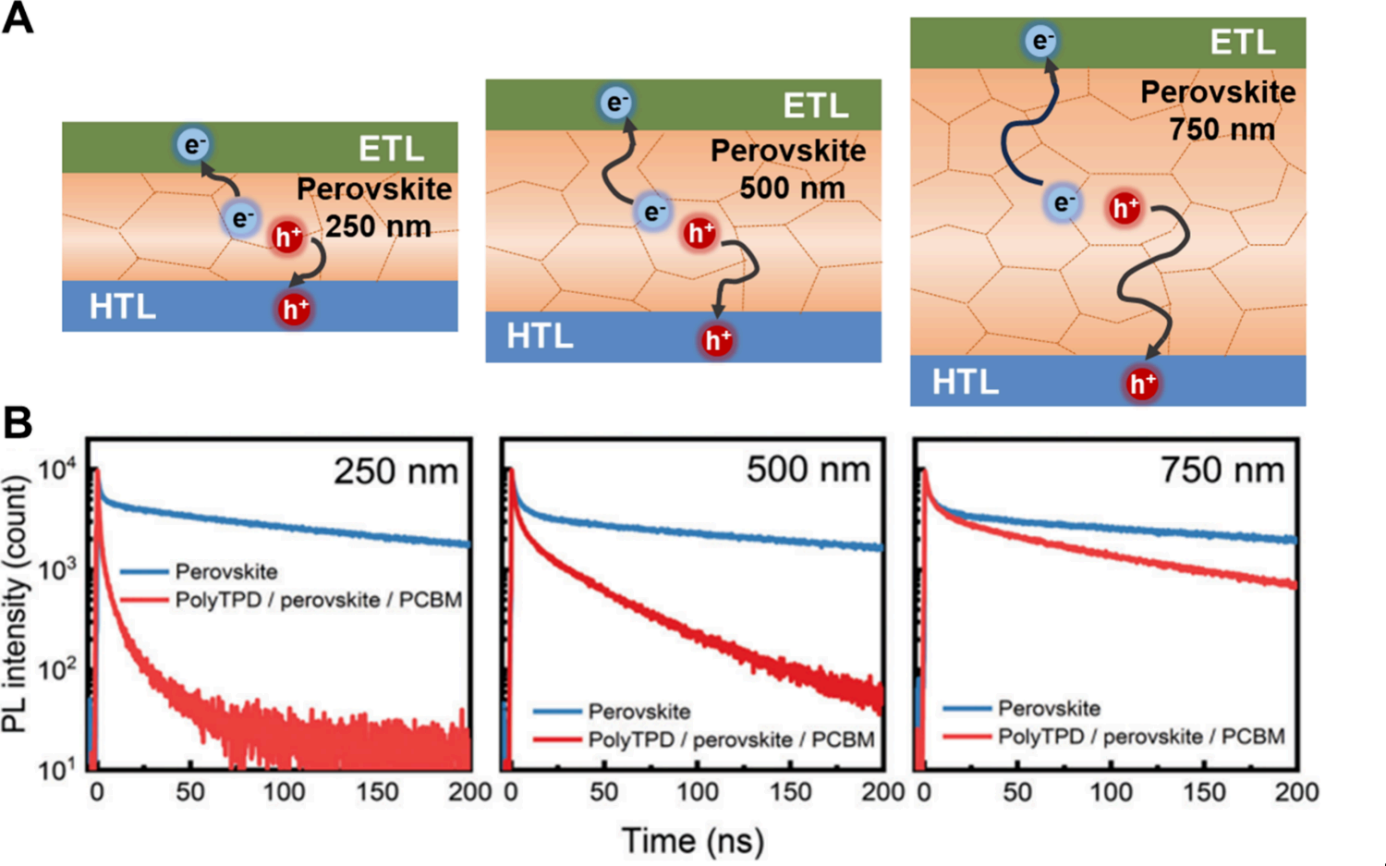
(A) Schematic representation of the effect of perovskite layer thickness (250, 500, and 750 nm) on charge carrier extraction. (B) TRPL decay curves of perovskite films with different thicknesses (250, 500, and 750 nm) with and without CTLs. Reproduced from ref (60) with permission from the Royal Society of Chemistry, 2020.

Although easy to ignore, the perovskite film thickness can influence charge carrier diffusion and extraction to the CTLs. Following bandgap excitation, charge carriers diffuse within the perovskite layer to the CTLs and subsequently toward the collecting electrodes. The maximum diffusion length of charge carriers in perovskite-based materials is reported to be on the order of micrometers, which is determined by both the lifetime of free carriers and the thickness of the perovskite layer.^58^ In thick perovskite films (Figure 2A) a longer diffusion length of the charge carriers has to be considered compared to thin perovskite films before charge carriers are extracted. This can result in slower decay observed on the TRPL traces compared to thin films (Figure 2B). Previous studies have shown that the charge carrier transfer rate decreases as the perovskite thickness increases.^42,59,60^ The PL decay curves of neat perovskite films (without CTL) with different thicknesses show no noticeable difference, indicating consistent film properties that probe intrinsic carrier recombination rates. In stark contrast, when CTLs (e.g., polyTPD as HTL and PCBM as ETL) are used, a perovskite layer thickness dependent PL decay was observed (Figure 2B), where the fastest decay was seen for thin perovskite films.^60^ Combining spectroscopy and diffusion modeling analysis, it was demonstrated that both diffusion and interfacial charge transfer are important in dictating the overall hole transfer rate.^42,59^ It is thus recommended to specify perovskite layer thickness while discussing charge transport rates and kinetic data analysis. However, care should be taken when presenting kinetic analysis on thin films (<100 nm), as surface traps are likely to dominate the charge carrier dynamics.^42^

The texturing of back contacts can also be important, as textured CTLs can increase the contact area and lead to higher internal light-scattering effects. Thus, the textured morphology of the charge transport layer dictates the photon escape probability from the perovskite films. Further complications may arise, if one encounters photon recycling^61,62^ effects when trapped photons are reabsorbed and re-emitted by the perovskite films multiple times.^63^

The photon recycling process thus can alter the carrier distribution within the perovskite film and affect the observed PL quantum efficiencies and lifetimes.^64,65^ For example, by simply cleaving MAPbBr_3_ single crystals and reattaching them with an insulating rough PMMA layer, a PL lifetime enhancement is seen, due to photon recycling.^65^ Apart from influencing light reflection processes, the substrate can also influence the crystallization of the perovskite layers. This in turn can affect the grain and interfacial defect properties of the assemblies and thus influence charge carrier extraction.^57^ Care must thus be taken to account for texturing of charge extraction layers and their potential impact on the measured carrier lifetimes, especially when compared to other literature examples.

## Time-Resolved Photoluminescence Measurements

For materials in which charge carriers can radiatively recombine, TRPL spectroscopy can be employed to monitor the relaxation kinetics of the excited state. The results obtained from TRPL spectroscopy are often used to interpret efficiency differences among devices (e.g., solar cells, LEDs) using different CTLs.^66^ Modeling analysis can assist in determining charge carrier diffusion lengths and carrier transfer yields in the presence of CTLs. Steady state PL spectroscopy is also a commonly used technique to show the influence of the CTL, as the observed quenching of the PL signal can be attributed to the charge transfer from the perovskite to the CTL. However, steady-state PL intensity measurements are highly prone to even slight changes in sample positioning, which can lower the amount of light collected. This is especially apparent in weakly emitting samples, which can yield erroneous results when compared between samples. On the other hand, TRPL measurements are less sensitive to slight positioning errors, yet have their own parameters that must be controlled. During TRPL measurements, parameters related to the excitation source (fluence, wavelength, and frequency) and the experimental setup (excitation direction, duration of exposure, detection time window, and sample atmosphere) strongly influence the accumulated emission–time profiles. TRPL measurements are often carried out by applying nanosecond laser pulse excitation. The limitation of pulse width fails to account for contributions from subnanosecond processes. The excited state lifetimes derived from PL measurements often differ from those obtained from ultrafast TA measurements, because of the variation in excitation laser pulse width, detection time frame, and nonexponential decay. Another aspect is that the experiments are performed in a comparative manner, where multiple samples (e.g., glass/perovskite and CTL/perovskite) are analyzed with one set of fixed parameters. This practice makes data interpretation ambiguous, as the relative change in the emission decay neglects other instrument parameters. It must be ensured that the effect of multiple instrument parameters is understood, and comparisons between samples are made under relevant conditions. A summary of these parameters and their effects are shown in Table 2. In the following sections, we explore how these experimental parameters can affect the measured excited state lifetimes and carrier injection rates.

**Table 2:** Experimental Parameters of TRPL Measurements and Their Effect on the Different Photophysical Processes in CTL/Perovskite Assemblies

excitation fluence	excitation wavelength	excitation direction
trap state filling	hot carrier population	fast decay from perovskite/CTL side
influence band-to-band charge carrier recombination	penetration depth	disentangle diffusion effects
influence charge extraction	density of mobile ions caused by produced phonons	
induce charge accumulation		
induce Auger recombination at high fluence		

excitation frequency	illumination time	measurement atmosphere
trap state depopulation	trap state filling	oxygen curing effect
influence band-to-band charge carrier recombination	photoluminescence brightening	degradation
influence charge extraction	ion migration caused by band bending change	

## Excitation Fluence

In perovskite films, most photophysical processes show a strong fluence dependence (i.e., number of generated charge carriers).^67−69^ The decay kinetics of the initial photogenerated charge carrier population can be analyzed considering a combination of first-order processes (e.g., recombination at trap states, extraction with CTLs), a second-order charge carrier recombination (e.g., band to band recombination), and a three-body recombination (e.g., Auger recombination) process, using eq 1
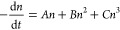
1where *n* is the photogenerated charge carrier density at time *t* following the excitation and *A*–*C* denote first-order, second-order, and third-order recombination rate constants, respectively.^70,71^ Importantly, the excitation intensity used in a TRPL measurement (and other time-resolved measurements) can influence the relative contribution of first-, second-, or third-order processes. Accurate modeling can be performed when considering the Shockley–Read–Hall (SRH) or extended SRH (SRH+) models, where the contribution of electrons and holes are considered separately. The SRH+ model can be described using eq 2
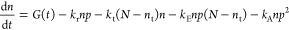
2where *G*(*t*) is the photogenerated carrier density per second, *n* is the free electron density, *n*_t_ is the trapped electron density, and *N* is the trap state density. *k*_r_ represents radiative recombination, *k*_t_ electron trapping, *k*_E_ Auger-assisted electron trapping, and *k*_A_ Auger-assisted electron–hole recombination rate constants. This extended model can be simplified to the SRH model by substituting *k*_E_ = 0 and *k*_A_ = 0. In a similar manner, if the density of trapped electrons equals 0 (*n*_t_ = 0), then *n*(*t*) = *p*(*t*) and the simplified *ABC* model (described in eq 1) can be applied. The benefit of using the SRH models is that trap-filling and/or Auger-trapping processes can be included in the decay kinetic model.^14^

Under low excitation fluence conditions, the contribution of radiative recombination (quadratic dependence on charge carrier density) dominates over nonradiative recombination (linear dependence on charge carrier density). In this situation, the number of available traps is not changed significantly. When the excitation intensity is increased to such an extent that the number of photogenerated carriers surpasses the number of trap states in the film, trap states can be saturated and become less dominant, further increasing the emission yield. Trap filling results in an increased emission yield up until a point at which a higher order Auger recombination pathway reverses the trend.^69^ Additionally, the gradual saturation of trap states can lead to the disappearance of the fast decay component.^72^

**Figure 3:**
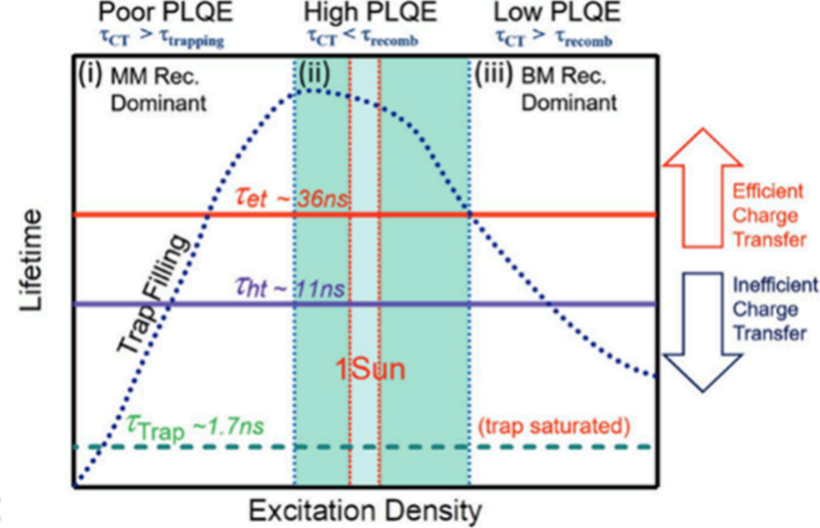
Effect of excitation fluence on the charge transfer and trap filling processes that are reflected in the PL lifetime. Reproduced from ref (73) with permission from Wiley-VCH, 2018.

The fast charge recombination process that becomes competitive with interfacial electron/hole transfer processes in CTL/perovskite assemblies can directly influence the analysis of the charge extraction kinetics. The influence of the excitation intensity can be seen from the charge injection yields for different CTL/perovskite assemblies. For example, a decrease in the injection yield was shown for both TiO_2_/MAPbI_3_ (from 95% to 10%) and MAPbI_3_/spiro-OMeTAD (from 99% to 50%) with increasing excitation intensity.^22^ In another example of PC61BM/perovskite and PEDOT:PSS/perovskite interfaces, efficient transfer was observed only under intermediate excitation conditions (∼1 sun irradiation).^73^ At low excitation intensities, the trap states in perovskite layers were active and not yet saturated, thus allowing charge carrier trapping to compete with the charge extraction process (as shown in Figure 3). Conversely, at higher excitation intensities, bimolecular recombination dominated, directly competing with the interfacial charge transfer.^73^ Due to the relatively slow charge mobility in the CTLs, compared to perovskite layers, transferred charges can accumulate at the CTL/perovskite interface. Simulations showed that accumulated charges have a nonlinear effect on transient PL. These accumulated charges decrease the transfer current but increase the interfacial charge carrier recombination current.^74^

## Excitation Wavelength

By tuning the excitation wavelength, the excess energy of the photogenerated charge carriers (hot electrons and hot holes) can be influenced (i.e., when using above bandgap excitation). Usually, hot charge carriers rapidly relax to the conduction band edge within ∼1 ps.^11^ The excess energy, however, can be utilized through carrier extraction at the interfaces where a potential barrier exists. This barrier—that disrupts carrier extraction and causes carrier accumulation—can be either caused by a conduction band mismatch or by the presence of trap states at the CTL/perovskite interface. Contrary to the expected quenching of the steady state PL signal, if carrier buildup occurs at the interfaces, an enhancement of the PL intensity and lifetime can be observed.^25,69^ In such cases, only hot carriers can traverse through the CTL/perovskite interface, resulting in a strong excitation energy dependence of the PL decay kinetics. For example, at defective compact TiO_2_/MAPbI_3_ interfaces, when the excitation energy was increased from 2.1 to 3.1 eV, an enhancement of the PL intensity was found compared to the glass/MAPbI_3_ interface. This enhancement was attributed to the suppression of electron accumulation at the interface, due to improved hot-carrier extraction.^25^ The excitation wavelength also controls the density of mobile ions in the perovskite films.^29^ The hot carriers, if not extracted, can cool to the respective band edges while transferring their excess energy to lattice vibrations (phonons). Therefore, a rapid decrease of the hole transfer rate with increasing excitation energy can be achieved. This is the result of decreased band bending under high-energy excitation.^29^

By variation of the excitation wavelength, it is possible to localize the excitation region within the perovskite film. In conventional perovskite absorbers, a 637 nm laser pulse has a penetration depth of ∼200 nm, while it is only ∼30 nm for a 405 nm laser pulse (also demonstrated for TA/TR in later sections). The absorbance difference of the film at 637 and 405 nm restricts the penetration depth. The difference in charge carrier generation across the perovskite film in response to excitation wavelength directly influences charge carrier diffusion/migration from the excited region to the CTL/perovskite interface.^42^

## Excitation Direction

**Figure 4:**
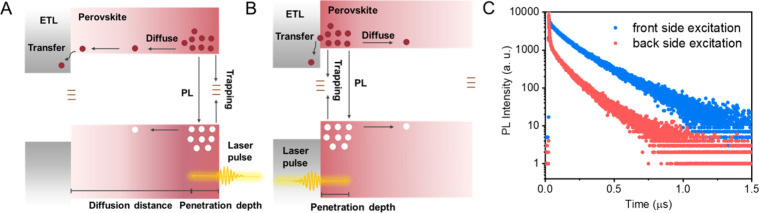
Schematic representations of (A) front-side and (B) back-side illumination of CTL/perovskite architectures, illustrating the relevant processes that dictate the PL decay. (**C**) PL decay curve of a TiO_2_/perovskite (500 nm, FA_0.83_Cs_0.17_Pb(I_0.83_Br_0.17_)_3_) sample, performed with different excitation directions with 460 nm laser wavelength. Our own unpublished data.

Front and back illumination of the sample can deliver different kinetic information. When partial device stacks consisting of perovskite films deposited on a CTL (ETL or HTL) are excited from the perovskite side (front side, Figure 4A), the photogenerated charges must first diffuse across the perovskite layer before charge transfer. In contrast, excitation from the CTL side (back side, Figure 4B) will generate charge carriers in the vicinity of the CTL, removing carrier diffusion from the picture. These two scenarios can be visualized in Figure 4C. An immediate fast decay is observed when back-side excitation is used, signaling that charge transfer is much faster than the charge carrier diffusion process.

Another factor that can influence the decay kinetics is the surface trap density of the perovskite, which varies from front to back as it experiences different surroundings. This aspect of trap density variation with different contact regions is often overlooked while estimating the kinetics of charge carrier recombination. For perovskite films deposited on a glass slide (i.e., in the absence of charge transfer) the observed PL lifetime is shorter when subjected to back-side illumination. This increase in the PL decay rate reflects faster surface charge carrier recombination at the back interface. Interestingly, if one coats the perovskite film with a noninteracting polymer such as PMMA, the PL lifetimes remain unchanged under front- and back-side excitations. These studies show the importance of interfacial defects which if ignored can directly affect the analysis of charge extraction.^75^

## Excitation Repetition Rate

**Figure 5:**
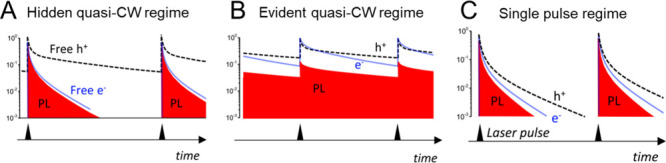
Illustration of PL decays in the (A) hidden and (B) evident quasi-continuous wave (CW) regime and (C) single-pulse excitation regimes. Reproduced from ref (14) with permission from Springer Nature, 2021.

In both TA and TRPL measurements, one employs a pulsed laser whose repetition rate can influence the excited state decay kinetics especially when long-lived trap states exist. The slow depletion rate of trap states becomes problematic when conducting TRPL measurements. This process has been studied in relation to solar cells to reliably conduct efficiency measurements, especially on slowly responding systems.^76^ A slower recovery after the laser pulse excitation can become problematic if the changes are sustained over a period of micro- to milliseconds.^77^ This problem becomes severe when performing measurements with high repetition rate (1–10 MHz) light pulses, as incomplete trap state depletion/recovery between the two pulses can introduce residual effects (Figure 5A,B). If we fail to allow sufficient time for a “dark recovery” cycle (viz., the time interval between excitation pulses), then the excited state dynamics of perovskites can lead to erroneous results. Increasing the time interval between excitation pulses gives trap states sufficient time to depopulate, resulting in a greater number of unoccupied trap states at the end of the cycle. As a result, when the laser pulse rate is reduced, newly generated free charge carriers encounter a higher density of vacant trap states, thus providing a better estimate of the excited state decay kinetics (Figure 5C, single pulse excitation regimes).^14,77^

When CTLs are interfaced with a perovskite film, one can also observe a similar dependence on the excitation frequency. When MAPbI_3_ was interfaced with both ETL (PCBM) and HTL (Spiro-MeOTAD and P3HT), an increase in the PL lifetime was observed with increasing excitation frequency. This was related to the ineffective charge capture as interfacial traps remained filled under the experimental conditions.^72^ Any excitation frequency dependent phototransformation, such as photoinduced phase segregation in mixed halide perovskites, can also introduce additional complexity in the data analysis.^78^ Thus, it is crucial to select a repetition rate that is slow enough to ensure that the PL lifetime is not being affected by residual trapped charge carriers. It is recommended that a range of repetition rates be tested to ensure that the PL lifetime is not dependent on the laser pulse frequency of the excitation.

## Light Soaking (Brightening, Darkening Effects) and Environment

Steady state or pulsed light irradiation is known to cause changes in the perovskite surface, as it can remediate the trap states and lead to PL brightening.^79−83^ In addition to improving the PL intensity, brightening can also prolong the PL lifetime (ref (86)).^83^ This effect is more pronounced for defect-rich (poorly performing) perovskite layers. Under continuous or quasi-continuous illumination, light soaking induced ion migration can further fill trap states^84,85^ and build up an interface barrier.^86,87^ For mixed halide perovskites, photoinduced phase segregation can shift the PL emission peak and change the PL lifetime of the system.^88,89^ In the case of the spiro-MeOTAD/perovskite assembly, the hole transfer rate was greatly affected by the illumination time. Light induced ion migration leads to a decreased band-bending, which is less favored for the hole transfer process compared to the initial band configuration.^29^

The atmosphere (e.g., vacuum/N_2_ vs air equilibrated) in which the light soaking experiment is carried out can also affect the PL intensity and lifetime of perovskite films. The exposure to dry air or oxygen during PL measurement can result in an increase in the PL intensity and lifetime.^90^ This brightening process is reversible, which signals that the underlying mechanism should be related to a photoactivated weak chemical interaction between O_2_ and the perovskite.^81,83,90−92^ Additionally, O_2_ is a well-known scavenger of electrons and can readily form O_2_^–^; thus, long-lived electrons in halide perovskites can be susceptible to this additional excited state process and potentially lead to perovskite degradation.^93^ In extreme cases, selective scavenging of electrons can lead to a buildup of holes, which can induce iodine oxidation in the perovskite lattice^4^ and cause phase segregation.^94^ Although high amounts of water accelerate the degradation process of perovskite layers, a small concentration of water introduced under controlled conditions exhibits a curing effect of defect states.^90^ Clearly the interactions with O_2_ and H_2_O can cause several different complicating effects, depending on the amount of each that is present. In general, it is recommended to perform time-resolved spectroscopy measurements under conditions that mirror what will be seen in devices—e.g. with minimal O_2_/H_2_O exposure (which is often achieved by encapsulation), and not under ultrahigh-vacuum conditions which lead to unique effects.^91^ Note that although we are discussing atmospheric effects with regard to TRPL measurements, the same considerations also hold for TA/TR measurements as well.

## Evaluation of PL Decay Curves

**Figure 6:**
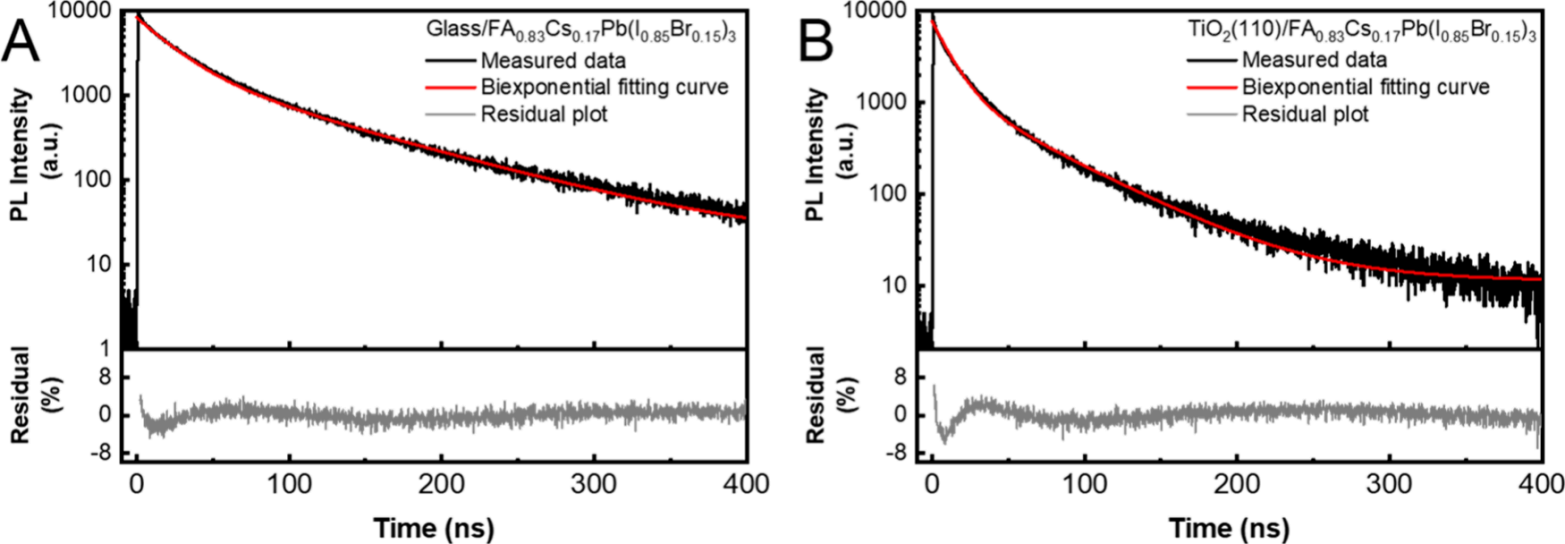
(A) Representative TRPL decay curves graphed together with the fitting curve and the residual of biexponential fitting of glass/perovskite (FA_0.83_Cs_0.17_Pb(I_0.83_Br_0.17_)_3_) and (B) biexponential fitting of TiO_2_ (110)/perovskite (FA_0.83_Cs_0.17_Pb(I_0.83_Br_0.17_)_3_). The excitation of the samples was at 467 nm, and the decay traces were monitored at 750 nm. Extended data from ref (95).

Different kinetic analysis protocols are employed to extract charge transfer rate constants from TRPL decay curves, such as (i) exponential and multiexponential fitting, (ii) stretched exponential fitting, (iii) modeling and global fitting, and (iv) use of the effective lifetime and solving the rate equation. Fitting the PL decay curves with a multiexponential function remains a popular approach because of its convenience in obtaining a good fit by increasing the number of exponential components. When a perovskite layer is deposited on CTLs, the long-lifetime component in a triexponential fit described by eq 3 is generally attributed to charge carrier recombination (band-to-band) and the middle component to recombination through trap states, while the fast components is related to the charge transfer to the CTL
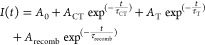
3where *I*(*t*) is the PL intensity at time *t*, *A*_0_ is the amplitude related to undecayed intensity within the time frame of the measurement, *A*_CT_ and τ_CT_ are the amplitude and lifetime of the charge transfer process, *A*_T_ and τ_T_ are the amplitude and lifetime of recombination through trap states, and *A*_recomb_ and τ_recomb_ are the amplitude and lifetime of recombination processes, respectively. When TRPL measurements of perovskite films in the presence of CTLs are employed, the middle component (arising from the contribution of trap states) is commonly not considered during kinetic analysis. To observe how this intermediate step influences the extracted charge transfer rate, we performed both bi- and triexponential fitting of the same data set. A representative TRPL data set and the fitted curve are shown in Figure 6. We used a glass/perovskite sample as the reference sample to obtain charge carrier recombination kinetics in the absence of an electron extraction layer.

The charge transfer rate constant (*k*_CT_) can be simply calculated by taking the inverse of the observed lifetime (eq 4):
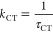
4To avoid the use of an arbitrary (and physically meaningless) number exponential function, the charge transfer lifetime can be calculated from the effective lifetime of the PL curves by using the charge transfer (CT) equation (eq 5). The benefit of using the effective lifetime is that it is model independent upon using an adequate fit. For this, a suitable reference sample must be used, that is assumed to have properties similar to those of the CTL/perovskite assembly under investigation.
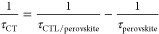
5When significant deviations are observed from the ideal exponential behavior (as in eq 2), the use of a stretched exponential function as described by eq 6 can be employed to analyze the PL decay. This evaluation method includes spatial inhomogeneities in the films, and all these processes can be described by a distribution of the rate constant.^96^ The deviation from ideality is described by a β parameter (0 < β < 1):
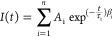
6To showcase the importance of the chosen data evaluation method, we determined the charge transfer rate constant for a TiO_2_/perovskite data set with the previously outlined methods. Other representative TRPL traces (triexponential and stretched exponential fitting), together with the fitted curve, are shown in Figure S1. Table 3 compares determined lifetimes and corresponding charge transfer rate constants using different kinetic analyses. Apparently, for the same set of data, a range of 3.4–26.3 ns was obtained for the charge transfer process. This degree of uncertainty shows why such a spread of electron transfer lifetime/rate constant exists in the literature values (Table 1). A critical factor in the determination of lifetime values is the number of exponents used for the kinetic fitting procedure. It is important to validate evaluation methods carefully so that each component is associated with a physical process, considering appropriate background/control measurements.

**Table 3:** Summary of TRPL Data Evaluation Approaches, with a Detailed Description of the Fitting Method Used, the Extracted Charge Transfer Lifetime, and a Literature Example Where the Method Was Used^a^

fitting protocol	τ_CT_ (ns)	rate constant (10^7^ s^–1^)	ref
biexponential fitting of PL decay traces; using the glass/perovskite sample as a reference and solving the CT equation	26.3	3.8	(97)
biexponential fitting of PL decay traces; directly attributing the shortest lifetime to charge extraction	11.2	8.9	(42)
triexponential fitting of PL decay traces; directly attributing the shortest lifetime to charge extraction	3.4	29.4	(86)
using a stretched exponential function to describe carrier diffusion processes within the perovskite films; directly attributing the shortest lifetime to charge extraction	11.6	8.6	(98)

a The fitting curves are shown in Figure S1.

As discussed above, the PL decay of perovskite films (in the absence and in the presence of CTL) is multiexponential. Before assigning each time constant of the multiexponential decay it is important to carry out a few checks: (i) control measurements of reference sample measurements on samples need to be performed to determine the contribution of trap states to the PL decay, (ii) complementary measurement techniques must be employed (e.g., PLQY measurements transient absorption or microwave conductivity measurements) to gain additional insights on the fate of photogenerated charge carriers, and (iii) front- and back-side excitations of perovskite films deposited on a glass slide (reference) and glass/CTL (sample) need to be performed to evaluate the contribution of trap states at different interfaces to the PL decay. Pulse frequency and excitation power dependent PLQY measurements coupled to a TRPL kinetic analysis can further minimize the error in lifetime analysis of the PL decay.^14^

## Transient Absorption/Reflection (TA/TR) Spectroscopy

**Figure 7:**
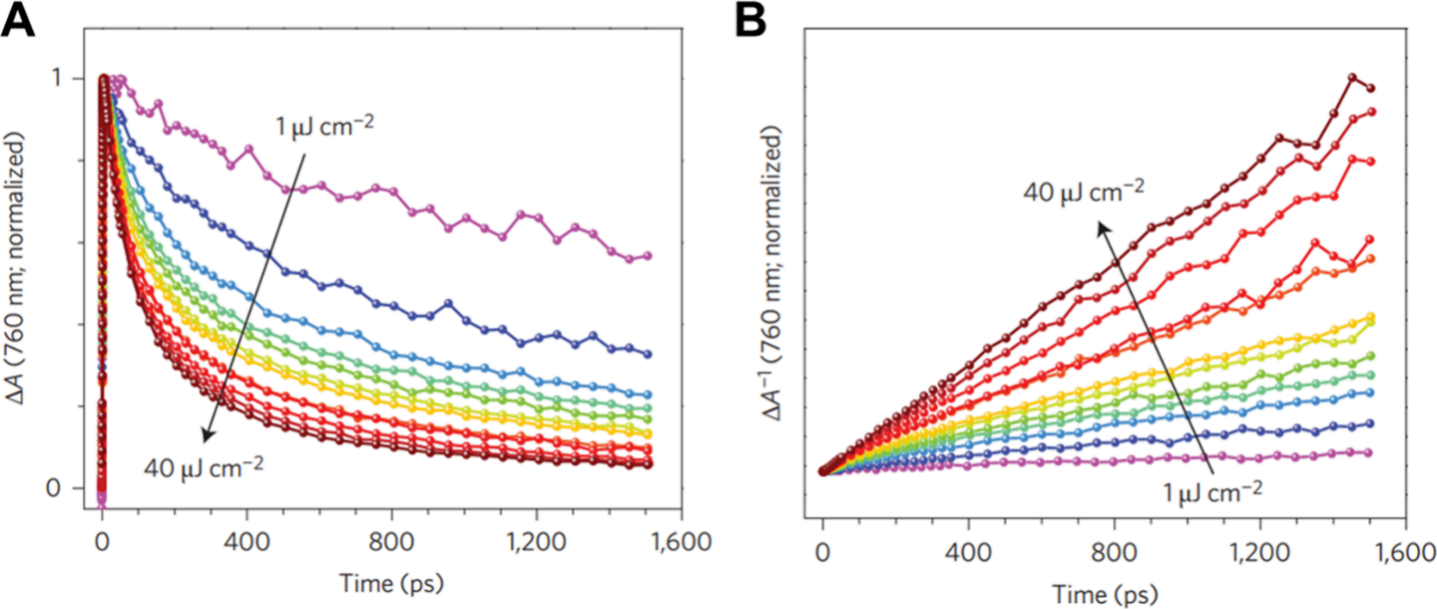
(A) Kinetic profiles of ground state bleach recovery at various pump intensities of MAPbI_3_ perovskite films deposited on mesostructured Al_2_O_3_ substrates. (B) Reciprocal of kinetic traces shown in (A), normalized at the maximum bleach (minimum Δ*A*^–1^). Reproduced from ref (70) with permission from Springer Nature, 2014.

In contrast to TRPL measurements, pump–probe TA/TR measurements can be used to directly monitor charge carrier decay via radiative and nonradiative pathways. 3D metal halide perovskites when subjected to bandgap excitation produce free carriers, as these materials inherently possess small exciton binding energies.^99,100^ The decay of free charge carriers in perovskites can be monitored through the recovery of the characteristic ground state bleach signal of the TA spectrum. Pristine perovskite films, which exhibit bimolecular charge carrier recombination, show laser intensity dependent decay, as the initial charge carrier concentration determines the overall lifetime of the excited state. Although the kinetic traces appear to be multiexponential (Figure 7A), they can be readily analyzed with a second-order kinetic fit at all laser intensities
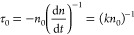
7where τ_0_ is the characteristic bimolecular recombination lifetime, *n*_0_ is the carrier density, and *k* is the second-order recombination rate constant (Figure 7B).^70^ A strong linearity of Δ*A*^–1^ versus time indicates good agreement with a bimolecular recombination mechanism.

The acceleration of the relaxation of the excited state can signal (i) the involvement of trap states, (ii) the presence of higher order carrier recombination processes, or (iii) carrier extraction in the presence of CTLs. The analysis poses a complex scenario if both hot and thermalized carriers compete for the electron transfer to CTL. Signatures of hot carrier extraction are often analyzed from the fast (subpicosecond) decay kinetics and from the broadened TA/TR spectrum with transient absorption in the higher energy region.^26^ Hot carrier extraction which occurs on the ultrafast time scale (subpicoseconds) cannot be monitored using traditional TRPL measurements, e.g., those that use nanosecond pulsed lasers and time-correlated single photon counting detectors, because of the time constraints of the excitation pulse and detector. The extraction of thermalized carriers can occur over a wide range of time scales often extending into the nanoseconds. Therefore, the distinction between these two processes is not clear, which adds to the dispersion of the carrier extraction rate constants obtained by TA/TR, which is summarized in Tables S2, S4, and S6.

Apart from probing the recovery of the ground state bleach signal arising from the depletion of the exciton band in the visible range, one can also look for spectral fingerprints in the infrared region. For example, the capture of electrons in the ETL, such as TiO_2_,^22,30,101^ or holes in the HTL, such as PEDOT,^102^ can be observed through characteristic absorption bands in the infrared region. Interestingly, these measurements all reveal an ultrafast charge transfer process between the CTL/perovskite (sub-ps) and almost no carrier extraction at the longer time scales.^22,30,101,102^ The signal intensity in the IR region, however, is low, and consequently, these measurements are carried out at relatively high fluences. This can introduce higher order carrier recombination processes, which must be considered in the evaluation process.

Both sample properties and the different experimental conditions influence the charge transfer rate constants, as can be seen from the example of TiO_2_ as the CTL (Table S2). These TA/TR measurements produce data analyzed by different kinetic models, further complicating the interpretation of the data. The situation gets even more complex if we consider variation in sample preparation, excitation conditions, and the selection of the detection window. Such anomalies have resulted in reporting a wide range of charge carrier extraction rate constants for perovskite/CTL assemblies. Even when we exclude the hot-carrier extraction component from the kinetic picture, a large dispersion in the charge carrier extraction rate constants remains (Table S2, 40 ps to 50 ns). A similar large dispersion in the extraction rate constants can be observed for PCBM ETLs (Table S4, 11 ps to 3.6 ns) and for the spiro-MeOTAD HTL (Table S6, 0.7 to 17 ns) as well, where a slightly better consistency of sample and instrument parameters can be seen. Overall, the large dispersion is not just rooted in the different measurement parameters, experimental conditions, or sample preparation, but also arises from the kinetic analysis/interpretation of the data. The experimental conditions that influence the kinetics of charge extraction are summarized in Table 4.

**Table 4:** Experimental Parameters of TA/TR Measurements and Their Effect on the Different Photophysical Processes in CTL/Metal Halide Perovskite Assemblies

excitation wavelength	excitation direction	excitation fluence
hot carrier generation	faster decay from CTL side	generation of hot carriers
penetration depth	disentangle diffusion effects	charge accumulation at the interface
pump-push-probe TA can reveal barrier height		

## Transient Reflection (TR) Spectroscopy

When TA measurements are performed, the probe light passes through the thin-film sample in a transmission mode geometry. In this arrangement, the collected data will be dominated by the response of the bulk properties of the perovskite films (e.g., carrier diffusion). In stark contrast, TR measurements probe the diffusely reflected light from the sample surface (dominated by the photoinduced change of the refractive index). TR is especially useful to gather surface sensitive information, such as surface defect dominated processes and surface charge carrier recombination rates.^49^ The control of the film thickness and sample geometry becomes important if one is interested in probing the CTL/perovskite interface. Cross-comparison of these measurements for perovskite samples without CTLs can be used to (i) reveal the effect of surface trap states or (ii) determine the perpendicular charge carrier mobility of perovskite thin films.^103,104^ However, care must be exercised, as the microstructure of the perovskite films (light scattering) can distort the TA signal and contain contribution from specular reflectance.^105^ TR can also be used in the case of nontransparent samples, where the probe light cannot pass through the sample and inhibit TA measurements (operating in transmission geometry).

TR measurements on samples where the CTL is coated directly on the top of the perovskite allows monitoring of interfacial charge transfer dynamics.^106^ In such cases, it is important to consider carrier diffusion processes when interpreting the excited state dynamics of these systems.^107^ In a similar manner, temperature dependent TA/TR studies can be used to determine the activation energy of the charge transfer across the CTL/perovskite interface.^106^ After the achievement of interfacial charge transfer, the fate of the injected carriers to the CTLs is often overlooked. A portion of these carriers can diffuse away from the CTL/perovskite interface; however, they can also participate in back-carrier transfer or reverse relaxation processes. This can proceed either directly between the respective band edges (interfacial charge carrier recombination) or through intermediate trap states located in the bandgap of the CTLs (e.g., oxygen vacancies in TiO_2_).^108^ CTL/perovskite interfaces where severe back-recombination exists will ultimately result in a poor device performance.^59^

## Excitation Wavelength and Fluence

**Figure 8:**
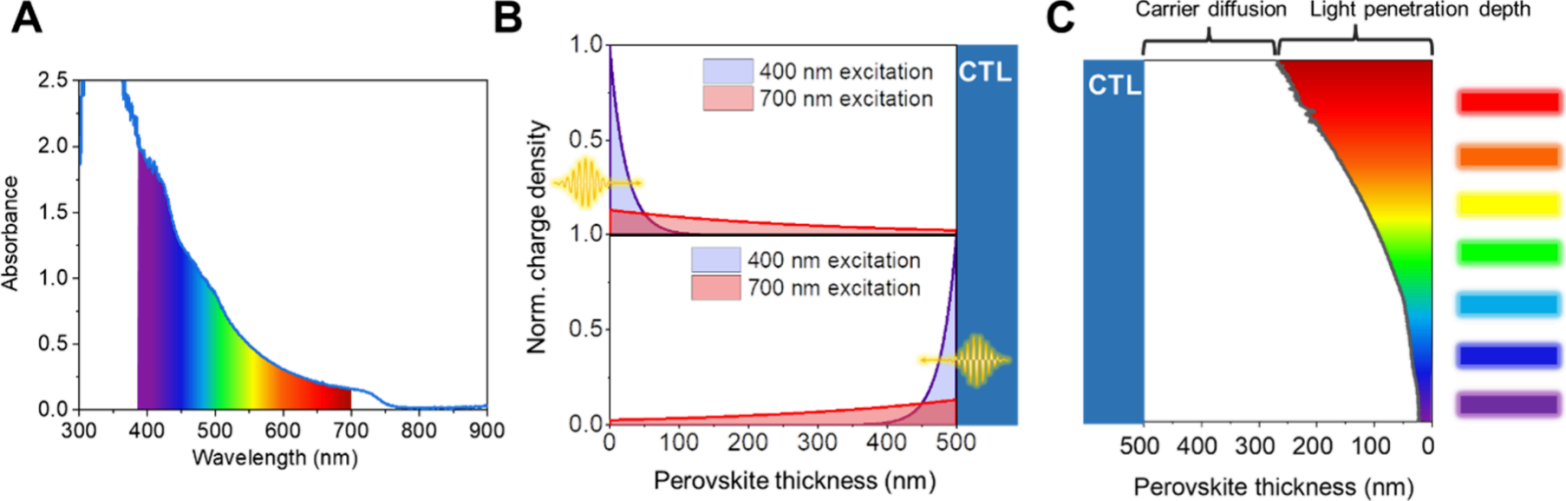
(A) UV–vis absorption spectrum of a 100 nm thick FA_0.83_Cs_0.17_Pb(I_0.83_Br_0.17_)_3_ perovskite layer on glass substrate. (B) Excited charge density distribution throughout a 500 nm thick perovskite film excited by 400 and 700 nm lasers: excited from perovskite/air interface (up) and excited from perovskite/CTL interface (down). (C) Scheme of light penetration conditions in the case of a 500 nm thick perovskite film excited by different wavelength lasers. Light penetration depth described as the penetration depth when incident light decays to  of its original intensity. Source: unpublished results.

In thin films, the choice of excitation wavelength is important since the penetration depth of the pump-beam can vary, causing a spatial distribution of photogenerated charge carriers and thus distorting TA/TR measurements. The excited charge density distribution (*G*) in the film right after excitation can be described by eq 8
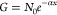
8where *N*_0_ is the photon flux at the surface, α is the absorption coefficient of the perovskite film, and *x* is the light penetration depth in the material. Figure 8A shows the UV–vis absorption spectrum of a 100 nm thick perovskite layer on a glass substrate. On excitation by a long wavelength laser (e.g., 700 nm), the deep penetration depth results in a uniform excited charge carrier population throughout the sample (Figure 8B,C). On the contrary, excitation with short wavelengths will result in a shallow penetration depth and inhomogeneous excitation. This will confine the excited volume to the near surface of the samples (Figure 8B,C), where charge carrier diffusion/migration to the CTL interface must be also included in the evaluation.^107^ When performing these experiments, with back-side excitation, where the perovskite volume close to the CTL/perovskite interface is excited, one can remove (or at least mitigate) carrier diffusion from the complex picture, allowing one to focus on charge injection (Figure 8B). This can be realized by physically rotating the sample^26^ or by inverting the device stack and depositing the CTL on top of the perovskite layer.^106^ When subjected to above-bandgap excitation, the probability of hot electron contribution increases significantly.^106^ Such excitation can favor hot carrier induced transfer at the CTL/perovskite interface, especially when thermalized carriers experience an energy barrier for the transfer.^30,109^

The charge carrier generation in perovskite films is directly proportional to the excitation intensity of the pump pulse. With high enough pump intensity (∼30 μJ cm^–2^), additional charge carrier recombination channels, such as the Auger process, become dominant, competing with the charge transfer processes and the bimolecular recombination in the perovskite layers.^30^ Since Auger processes accelerate the relaxation of the excited state, they can affect both the decay dynamics and the shape of the transient spectra. The high local electric field can cause the shift of the optical transition, resulting in the Stark effect.

Another effect of excitation fluence is manifested in the band-filling effect, which is usually observed through the broadening and blue-shifting of the bleach signal as charge carriers accumulate within the conduction and valence bands.^70^ At higher excitation intensities, the valence/conduction bands are filled to a greater degree, and as a result of the Pauli exclusion principle, this shifts allowable transitions to higher energies beyond the band edges. The carrier-induced change in the bandgap can be modeled according to eq 9(110)
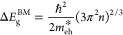
9where  is the change in the optical bandgap due to the Burstein–Moss band filling effect,  is the reduced effective mass (), and *ℏ* is the reduced Planck constant. The broadening and shift of the optical bandgap (and thus ground state bleach) due to the Burstein–Moss effect is readily apparent and can be easily measured by analyzing the fwhm of the bleach as a function of fluence.^70^ The threshold intensity (or carrier population, *n*) at which the shift occurs correlates well with the trap state density in perovskite thin films,^111^ and thus can be used to extract further information from a perovskite sample.

**Figure 9:**
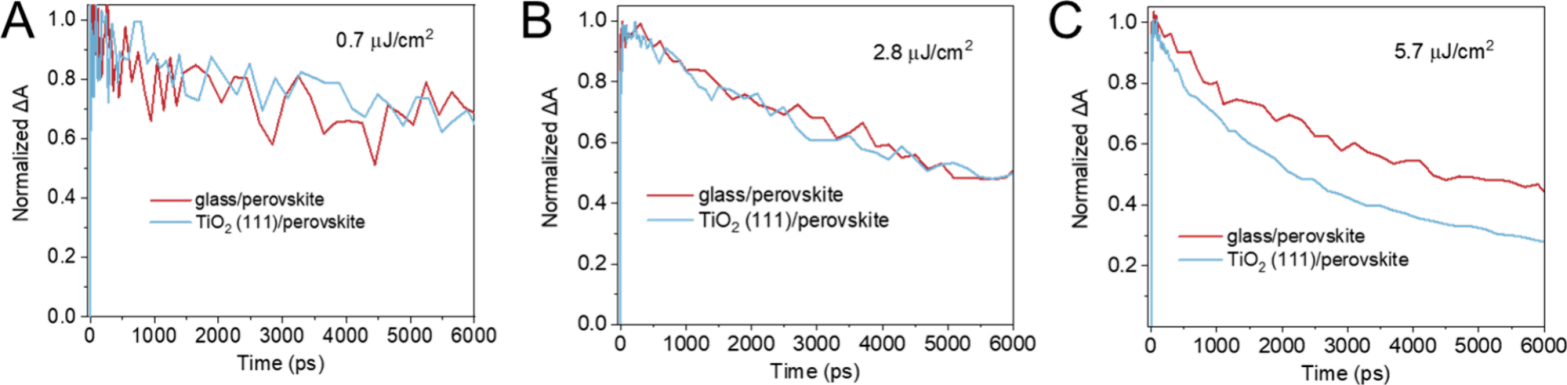
Bleach recovery profiles of TiO_2_ (111)/perovskite (FA_0.83_Cs_0.17_Pb(I_0.83_Br_0.17_)_3_) samples recorded following a 600 nm laser pulse excitation with pump fluences of (A) 0.7 μJ cm^–2^, (B) 2.8 μJ cm^–2^, and (C) 5.7 μJ cm^–2^. The decay profiles were extracted from the transient absorption spectra at 725 nm. Reproduced from ref (95) with permission from the American Chemical Society, 2024.

Excitation fluence dependent measurements can also be used to separate first-order processes (charge transfer and trapping) from second-order band-to-band charge carrier recombination. Furthermore, if an electric barrier exists for charge transfer at the CTL/perovskite interface,^112^ often the excitation wavelength or the fluence should be varied to overcome it. The latter case is shown in Figure 9 where at lower fluences no electron transfer can be observed in a TiO_2_/perovskite assembly (Figure 9A,B).^95^ As the excitation fluence increases further, electrons accumulate at the conduction band due to the band-filling effect. This electron accumulation can overcome the electric barrier at the CTL/perovskite interface and initiate electron transfer (Figure 9C).^95^

## Assessing the Merits of Kinetic Analysis

From the viewpoint of experimental parameters, if one aims to compare rate constants obtained from TRPL and TA measurements, then one must consider that there is often a difference between the excitation intensity for the two techniques. Generally, TA is carried out at higher excitation fluences than TRPL measurements (in order to generate small changes in absorption); thus, higher order carrier recombination processes can contribute to the relaxation of the excited state. A similar difference exists in the excitation frequency, where TRPL measurements are generally carried out at higher and easily tunable repetition rates compared to TA. As discussed previously, higher repetition rates can result in variation in whether long-living trap states are depopulated between measurements. Additionally, TRPL measurements typically use ns-pulsed lasers (in common TCSPC setups), whereas TA measurements that employ fs-pulsed lasers have orders-of-magnitude shorter pulse widths, further complicating comparisons.

As the strategies to conduct transient spectroscopy experiments and data analysis in TA/TR studies vary, one observes a wide dispersity in the measured lifetimes and charge transfer rate constants. Analyzing kinetic profiles probed at a single wavelength (usually the excitonic bleach signal) can bring some uniformity in obtaining a better understanding of charge carrier dynamics. The analysis of such kinetic traces can be carried out by either employing multiexponential fitting or by solving complex differential equations (analytical solutions to kinetic equations and numerical integration of rate equations adding charge diffusion and reabsorption processes). The simplicity of single-wavelength analysis can be overshadowed by the fact that it neglects the contribution of different components (e.g., defect states or higher order transitions) to the overall bleach signal.^21^ A further complication arises from the broad, often overlapping features observed in TA spectroscopy, which makes the separation of different components difficult. A complete picture of excited state interactions can only be gleaned if a detailed analysis of the transient response at different wavelengths is carried out.

**Figure 10:**
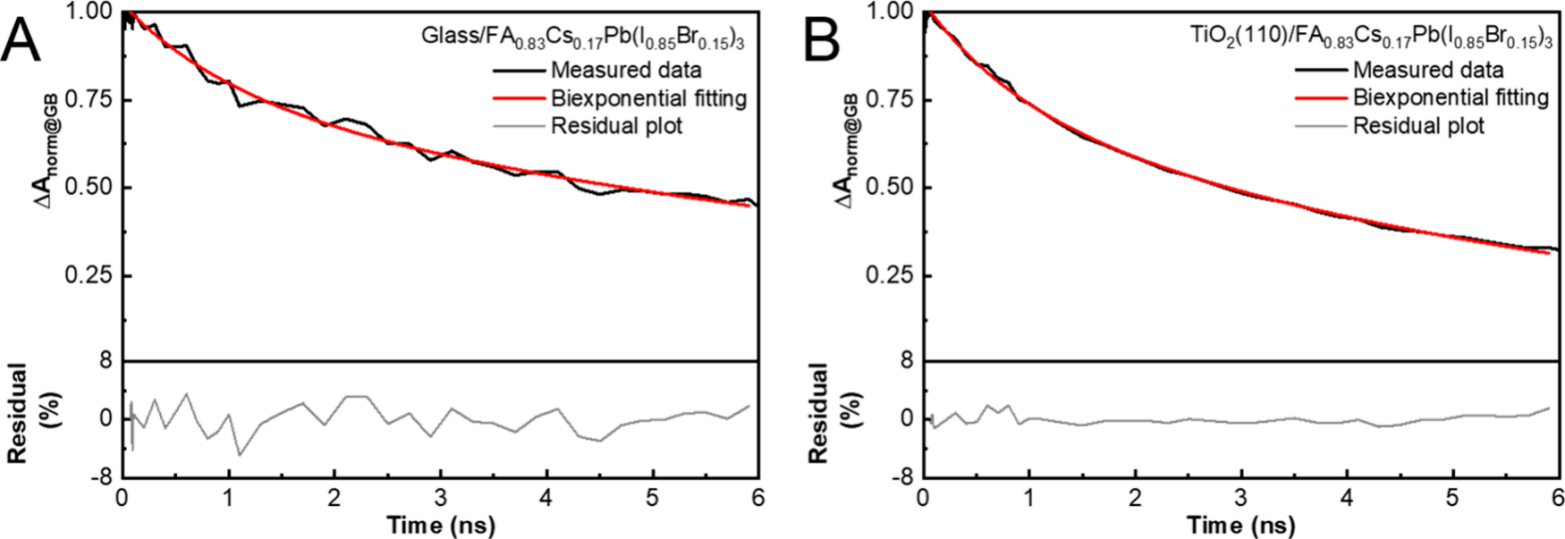
Representative TA decay curves graphed together with the fitting curve and the residual of biexponential fitting of (A) glass/perovskite (FA_0.83_Cs_0.17_Pb(I_0.83_Br_0.17_)_3_) and (B) TiO_2_ (110)/perovskite (FA_0.83_Cs_0.17_Pb(I_0.83_Br_0.17_)_3_). The measurements were performed following 600 nm laser pulse excitation with pump fluences of 5.7 μJ cm^–2^. The decay profiles were extracted from the transient absorption spectra at 725 nm. Extended data from ref (95).

Analysis of the transient absorption decay/recovery recorded in TA/TR studies is carried out in a way similar to that for the evaluation of TRPL data. Typically, one of the lifetimes from the multiexponential fit (usually fast time component) is considered to obtain information on the charge carrier extraction kinetics. The lifetimes of the fast components between the reference sample (e.g., on glass, without charge injection) and that of the CTL/perovskite are used to extract the charge transfer rate constant (similar to eq 4 used in TRPL analysis). The latter approach relies on the physicochemical similarity of the reference sample and the CTL/perovskite assembly. An example of multiexponential kinetic analysis is shown in Figure 10. Again, the lifetimes extracted from these experiments are strongly dependent on the data analysis method. Table 5 summarizes the different approaches employed to extract the lifetimes from the kinetic analysis. A dispersion of lifetimes in the range of 0.2–14.1 ns is shown, the large variation of which is also reflected in the determined electron (or hole) transfer rate constants.

**Table 5:** Summary of TA Data Evaluation Approaches, with a Detailed Description of the Fitting Method, the Extracted Charge Transfer Lifetime, and a Literature Example Where the Method Was Used^a^

fitting protocol	τ_CT_ (ns)	rate constant (10^9^ s^–1^)	ref
biexponential fitting of TA decay traces; using the glass/perovskite sample as a reference and solving the CT equation	14.1	0.07	(19)
biexponential fitting of TA decay traces; directly attributing the shortest lifetime to charge extraction	0.9	1.11	(116)
biexponential fitting of TA decay traces; directly attributing the longest lifetime to charge extraction	6.4	0.16	(117)
triexponential fitting of TA decay traces; directly attributing the shortest lifetime to charge extraction	0.2	5.00	(118)
using a stretched exponential function to describe carrier diffusion processes within the perovskite films; directly attributing the shortest lifetime to charge extraction	1.9	0.53	(22)

a The fitting curves are shown in Figure S2.

More complex options, such as global or target analysis, also exist to analyze transient decay that can overcome the shortcomings of only considering single wavelengths.^31^ When these methods are compared with fluence dependent measurements, a self-consistent charge transfer rate constant can be obtained for different CTL/perovskite systems. However, one should be cautioned that global analysis procedures, which were originally developed for more well-behaved molecular systems, do not always capture the complex excited state behavior of semiconductors such as halide perovskites.

Both the bleach recovery in the TA and emission decay in TRPL represent deactivation of the excited state due to charge recombination. Since the decay of the excited state follows a biexponential trend, the kinetic fits weigh in short and long components of the decay depending on the time scale of detection. As a result of this anomaly, TRPL and TA, can provide different kinetics of charge transfer depending on the time scale of detection. The values summarized in Tables 3 and 5 highlight the differences in reported values using these two detection techniques. Additional components in the charge carrier kinetics can be extracted with companion techniques such as time-resolved microwave conductivity measurements. In such cases long lifetimes in perovskite films extending up to microseconds can be observed.^113^ To simulate real-time scenarios one can also conduct spectroelectrochemical studies by probing the transient behavior under applied electrochemical bias.^114,115^

## Outlook—Merit of Spectroscopy Protocols

Solar energy conversion devices typically work under low irradiation fluence (i.e., AM1.5, 100 mW/cm^2^), whereas many time-resolved spectroscopy techniques use comparatively higher excitation intensities and pulsed excitation at a selected wavelength, making the correlation of the parameters related to charge transfer difficult. Furthermore, during steady state irradiation, the state of the solar cell operation indirectly influences the net charge transfer process. For example, under open circuit conditions, charges accumulate near the collecting electrode, thus limiting additional charge transfer. On the other hand, under short circuit conditions, the charges at contact electrodes are drained, thus accelerating charge transfer. Hence, one needs to exercise caution while correlating the charge transfer kinetics from time-resolved spectroscopy techniques to steady-state solar cell operation. Nevertheless, transient spectroscopy provides some useful information for selecting ETL and HTL layers and establishing the dependence of charge transfer on several chosen experimental parameters.

As discussed in this review, the estimate of charge transfer rate constants obtained from TRPL and TA/TR techniques is dependent not only on the detection limit but also on the sample preparation and thickness, geometry of excitation, excitation wavelength, fluence, and atmosphere. Additionally, the kinetic analysis used to interpret the transient decay traces can yield differing values. As a result, a broad range of charge transfer rate constant reporting has appeared in the literature. Whereas it is almost impossible to standardize the procedure globally, it is important for the scientific community to recognize the limitations of measurements, as well as the kinetic analyses employed to extract charge transfer rate constants. The following list presents *Best Practices* that the researchers in the field should adopt in their spectroscopic measurements.

(A) Clearly describe the sample preparation, including composition, grain size distribution, and the thickness of each layer (including interlayers/passivation layers).
(B) Provide excitation conditions, including geometry, fluence, excitation wavelength, and repetition rate.
(C) Include absorbance of the sample at the excitation wavelength and indicate whether the excitation is homogeneous throughout the sample.
(D) A broader model to account for ultrafast (fs–ns) as well as slow (ns−μs) components responsible for charge transfer should be adopted and not just tailored to a specific detection time window.
(E) A physical charge transfer model needs to be established first before attempting to analyze the kinetic traces. If possible, the reasons for ruling out other models need to be discussed.
(F) While the model is unsure, the average lifetime, which is model independent, can be analyzed to showcase charge transfer.

By reporting details of sample preparation, experimental procedure, and kinetic analysis, it should be possible to compare and validate kinetic results reported in the literature. Such a concerted effort will aid better understanding of the perovskite/charge transport layers and aid the development of perovskite photovoltaic and optoelectronic devices toward real-world applications.

## Supporting Information Available

Tables summarizing charge transfer rates from literature examples for different CTL/perovskite interfaces, together with relevant experimental parameters and evaluation methods used, and additional figures and tables showing various exponential fitting protocols (PDF)
