# Supplementary material for: Charge Transfer Kinetics in Halide Perovskites: On the Constraints of Time-Resolved Spectroscopy Measurements
Source: ACS Energy Lett. 2024 Jun 5;9(6):3187–203. doi: 10.1021/acsenergylett.4c00736 (PMC11190987; doi:10.1021/acsenergylett.4c00736)
Supplement: Supplementary file 1 — nz4c00736_si_001.pdf [file nz4c00736_si_001.pdf]

Supporting Information

Charge Transfer Kinetics in Halide Perovskites: On the Constraints of Time-Resolved Spectroscopy Measurements

Xiangtian Chen[1], Prashant V. Kamat[3], Csaba Janáky[1,2], Gergely Ferenc Samu[2,4,*]

[1]Department of Physical Chemistry and Materials Science, Interdisciplinary Excellence Centre, University of Szeged, Aradi Square 1, Szeged, H-6720, Hungary

[2]ELI-ALPS, ELI-HU Non-Profit Ltd., Wolfgang Sandner street 3., Szeged, H-6728, Hungary

[3]Department of Chemistry and Biochemistry, University of Notre Dame, Notre Dame, Indiana 46556, United States

[4]Department of Molecular and Analytical Chemistry, University of Szeged, Dóm Square 7-8. Szeged, H-6721, Hungary

Table S1: Summary of TRPL measurement parameters, used evaluation methods and extracted lifetimes related to electron transfer to TiO_2 layers from perovskite samples.

Architecture	Perovskite thickness	λ_{exc}/ nm	Fluence	τ_{CT}	Rate Constant (10^7 s^{-1})	Evaluation method	Comments	Reference
$TiO_2/MAPbI_3$	n.a.	625	<0.1 µJ cm^{-2}	11 ns	9.1	Use of rate equation in data analysis.	Effective lifetime is used in the CT equation. At low fluences high CT yields are observed. The CT rate is calculated from the low excitation regime.	1
TiO_2 single crystal/$MAPbI_3$	70 nm	60	0.63 µJ cm^{-2}	0.17 – 20.6 ns	4.9-588.2	Bi-exponential fitting.	Effective lifetime is used in the CT equation. Hot carrier extraction is not observed in the PL response of the films.	2
$TiO_2/MAPbI_3$	n.a.	40	n.a.	2.3 – 5.8 ns	17.2-43.5	Bi-exponential fitting.	Effective lifetime is used in the CT equation. The effect of TiO_2 rod length is studied in determining device performance.	3
$TiO_2/MAPbI_3$	600 nm	46	n.a.	2.1 ns	47.6	Tri-exponential fitting.	The fast component was attributed to the CT.	4
$TiO_2/MAPbI_3$	290 nm	640	n.a.	9.7 ns	10.3	Bi-exponential fitting.	The fast component was attributed to the CT.	5

Table S2: Summary of TA/TR measurement parameters, used evaluation methods and extracted lifetimes related to electron transfer to TiO_2 layers from perovskite samples.

Architecture	Perovskite thickness	λ_{exc}/ nm	Fluence	τ_{CT}	Rate Constant (10^9 s^{-1})	Evaluation method	Comments	Reference
c-$TiO_2/MAPbI_3$	65 nm	400	10 µJ cm^{-2}	370 ps	2.7	Multiexponential fit to single wavelength decay trace.	Slow lifetime component is correlated with CT. Longer lifetime with the TiO_2 ETL than for bare samples. This is attributed to a potential barrier at the interface. Only hot electron transfer is present here. 9% efficient solar cells are studied.	6
mp-$TiO_2/MAPbI_3$	n.a.	400	n.a.	260 – 307 ps	3.3-3.8	Multiexponential fit to single wavelength decay trace in the 10 ps – 2000 ps regime.	Fast component (middle) is directly correlated with the electron transfer time from the perovskite to the ETL. 8-10 % efficient solar cells are studied.	7
mp-TiO_2/graphene QD/$MAPbI_3$				90-106 ps	9.4-11.1			
mp-$TiO_2/MAPbI_3$	200 nm	460 (and 750)	2.0 µJ cm^{-2}	0.1 – 0.2 ps	5000-10000	Multiexponential fit to single wavelength decay trace at multiple wavelengths.	Complete solar cells were studied. The rise of the NIR response was correlated with electron injection. 13.5 % efficient solar cells are studied	8
c-$TiO_2/MAPbI_3$	n.a.	390 (and 600)	n.a.	39.9 ps	25.1	Multiexponential fit to single	Slow lifetime component was assigned to carrier	9
mp-$TiO_2/MAPbI_3$				89.6 ps	11.1			

c-TiO_2/mp-TiO_2/$MAPbI_3$				150 ps	6.7	wavelength decay trace.	transfer. The degree of PbI_2 passivation was also different for the architectures.	
c-TiO_2/$MAPbI_3$	100 nm	370	3.0 μJ cm^{-2}	<10 ps	>100	Multiexponential fit to single wavelength decay trace, lower wavelength than the GB.	Hot carrier extraction was studied in this case. Holes were extracted faster in the case of spiro-MeOTAD than electrons with TiO_2.	10
TiO_2 single crystal/$MAPbI_3$	70 nm	550 (and 600)	0.1 - 3.0 μJ cm^{-2}	30 (0.05), 70 (0.06) and 1420 (0.07) ps	33.3 (20000), 14.3 (16666.7) and 0.7 (14285.7)	Multiexponential fit to single wavelength decay trace.	The fastest lifetime component was assigned to CT. Hot carrier extraction was separately probed by monitoring the NIR response of the assemblies.	2
TiO_2/$MAPbClI_2$	n.a.	470	0.5 – 12.5 μJ cm^{-2}	1.8 ns	0.6	Monoexponential fit to single wavelength decay trace.	The average lifetime was compared to a reference sample and the difference was assigned to k_{ET}. Apart from fs-TAS also ns-TAS experiments were carried out.	11
mp-TiO_2/$MA_{(1-y)}FA_yPbI_xBr_{(3-x)}$	n.a.	620	<1.0 μJ cm^{-2}	100 fs	10000	Monoexponential fit to single wavelength decay trace.	Rising of the NIR part of the TAS is monitored.	12
c-TiO_2/$MAPbI_3$	400-500 nm	485 (and 595)	0.5 – 75.0 μJ cm^{-2}	50 ns	0.02	Global analysis of TAS data and determination of average lifetimes at different fluences.	The fluence dependence of the average lifetime was fitted to a linear function. The intercept is related to the carrier extraction rate, which was compared to a reference sample.	13
mp-TiO_2/$MAPbI_3$				9.1 ns	0.1			

Table S3: Summary of TRPL measurement parameters, used evaluation methods and extracted lifetimes related to electron transfer to PCBM layers from perovskite samples.

Architecture	Perovskite thickness	λ_{exc}/ nm	Fluence	τ_{CT}	Rate Constant (10^7 s^{-1})	Evaluation method	Comments	Reference
PCBM/$MAPbI_3$	290 nm	635	0.06-0.13 mW cm^{-2}	36 ns	2.8	Bi-exponential fitting.	The slow component was correlated with electron transfer. The slow component saturates as the fluence increases.	14
PCBM/$MAPbI_3$	750 nm and 250 nm	405	3 nJ cm^{-2}	1.4 ns	71.4	Bi-exponential fitting.	The fast component is correlated with charge transfer. To extract the CT rate the excitation was carrier out from the CTL side. The CT rate was found to be independent of the perovskite layer thickness. Full device stack was explored in the study.	15
PCBM/$MAPbI_3$	600 nm	464	n.a.	1.3 ns	76.9	Tri-exponential fitting.	The fast component is correlated with charge transfer. PCBM was found to increase charge	4

							extraction, while TiO_2 showed blocking behavior.	
PCBM/$MAPbI_3$	65 nm	600	1.3 μJ cm^{-2}	0.4 ns	250	Mono-exponential fitting.	CT equation is used to extract CT rate. The extracted lifetimes are correlated with TAS measurements.	16

Table S4: Summary of TA/TR measurement parameters, used evaluation methods and extracted lifetimes related to electron transfer to PCBM layers from perovskite samples.

Architecture	Perovskite thickness	λ_{exc}/ nm	Fluence	τ_{CT}	Rate Constant (10^9 s^{-1})	Evaluation method	Comments	Reference
PCBM/$MAPbI_3$	65 nm	400	10 μJ cm^{-2}	370 ps	2.7	Multiexponential fit to single wavelength decay trace.	The slow component is correlated with CT. 10 % efficient solar cells were studied.	6
PCBM/$MAPbI_3$	65 nm	600	13 μJ cm^{-2}	0.4 ns	2.5	Multiexponential fit to single wavelength decay trace.	The fast lifetime component is correlated with CT. The determined lifetimes are correlated with results from TRPL measurements (that are monoexponential in nature)	16
PCBM/$MAPbI_3$	280 nm	400	7.6 μJ cm^{-2}	<1ps	>1000	Global fitting of the data.	A 20 ps lifetime component is assigned to carrier diffusion from the surface of the film to the bulk in thick films.	17
	60 nm			11 ps	90.9			
PCBM/$MAPbI_3$	110 nm	485 (and 600)	2.5 - 150 μJ cm^{-2}	3.57 ns	0.3	Global fitting of the data and band integral approach.	The first order time constant was used as the electron transfer rate. In the case of bare perovskite samples this resembles trap states on the surface of the samples. It is noted that it contains contribution from diffusion/migration processes. This was removed from the data by subtracting k values of a reference sample where no transfer would be expected.	18
PCBM/$MAPbI_3$	60 nm	388	25 μJ cm^{-2}	0.6 ps	1666.7	Global fitting of the data. The rise of the excited state absorption was monitored for the ETL and HTL.	Apart from the ultrafast electron injection in PCBM an ongoing electron transfer component is also shown in the 25 ps range.	19

Table S5: Summary of TRPL measurement parameters, used evaluation methods and extracted lifetimes related to hole transfer to Spiro-MeOTAD layers from perovskite samples.

Architecture	Perovskite thickness	λ_{exc}/ nm	Fluence	τ_{CT}	Rate Constant (10^7 s^{-1})	Evaluation method	Comments	Reference
Spiro-MeOTAD/ $MAPbI_3$	65 nm	600	1.3 µJ cm^{-2}	0.66 ns	151.5	Mono-exponential fitting.	CT equation is used to extract CT rate. The extracted lifetimes are correlated with TAS measurements.	16
Spiro-MeOTAD/ $(FAPbI_3)_{0.85}(MAPbBr_3)_{0.15}$	400 nm	470 and 640	0.4 W cm^{-2}	25 – 100 ns	1-4	Effective lifetime is extracted from diffusion equation and global fitting.	CT equation is used to extract CT rate. Continuous illumination is used to fill surface trap states. Shorter wavelength excitation provides better trap state passivation.	20
Spiro-MeOTAD/ $Cs_{0.055}FA_{0.80}MA_{0.145}PbI_{2.55}Br_{0.45}$	n.a.	405	200 mW cm^{-2}	1.0 – 2.1 ns	47.6-100	Multi-exponential fitting.	The fastest component is attributed to CT. Irradiation of samples increased the hole extraction rate, which was caused by ion migration in the samples.	21
Spiro-MeOTAD/ $Cs_{1-x-y}MA_xFA_yPbI_{3-z}Br_z$	135 nm	648	n.a.	0.9 ns	111.1	Bi-exponential fitting.	Slow component is linked to hole injection process.	22
Spiro-MeOTAD/ $MAPbI_3$	n.a.	625	<0.1 µJ cm^{-2}	1.8 ns	55.6	Use of rate equation in data analysis.	Effective lifetime is used in the CT equation. At low fluences high CT yields are observed. The CT rate is calculated from the low excitation regime.	1

Table S6: Summary of TA/TR measurement parameters, used evaluation methods and extracted lifetimes related to hole transfer to Spiro-MeOTAD layers from perovskite samples.

Architecture	Perovskite thickness	λ_{exc}/ nm	Fluence	τ_{CT}	Rate Constant (10^9 s^{-1})	Evaluation method	Comments	Reference
Spiro-MeOTAD/ $MAPbI_3$	65 nm	600	10 µJ cm^{-2}	0.66 ns	1.5	Multi-exponential fitting to single	The short lifetime component is correlated with the hole transfer rate. The extracted lifetimes are	16

						wavelength decay trace.	correlated with TRPL measurements.	
Spiro-MeOTAD/ $MAPbI_3$	200 nm	460	2.0 µJ cm^{-2}	0.75 ps	1333.3	Multi-exponential fitting to single wavelength decay trace at multiple wavelengths.	Complete solar cell was studied. The rise of the Nir response was correlated with hole injection. 13.5% efficient solar cell was studied.	8
Spiro-MeOTAD/ $MAPbI_3$	100 nm	370	3.0 µJ cm^{-2}	< 1ps	>1000	Multi-exponential fitting to single wavelength decay trace lower wavelength as the GB.	Hot carrier injection was studied in this case. Holes were extracted faster in the case of spiro-MeOTAD than electrons with TiO_2.	10
Spiro-MeOTAD/ $MAPbClI_2$	n.a.	470	0.5 – 12.5 µJ cm^{-2}	16 ns	0.06	Mono-exponential fitting to single wavelength decay trace	The average lifetime was compared to a reference sample and the difference was assigned to the k_{HT}. Apart from fs-Tas also ns-Tas experiments were carried out.	11
Spiro-MeOTAD/ $MAPbI_3$	400 - 500 nm	485 (and 595)	0.5 - 75 µJ cm^{-2}	16.7 ns	0.06	Global analysis of TAS data and determination of average lifetimes at different fluences.	The fluence dependence of the average lifetime was fitted to a linear function. The intercept is related to the carrier extraction rate, which was compared to a reference sample.	13

TRPL data evaluation

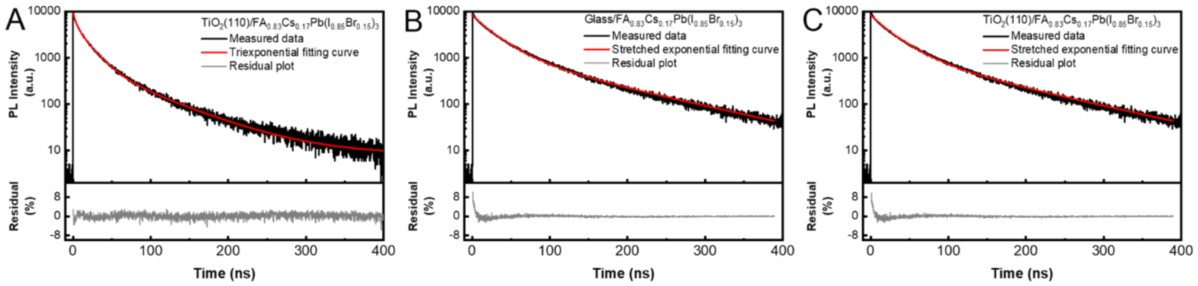

Figure S1: TRPL decay curves graphed together with the fitting curve and the residual of **A:** triexponential fitting of TiO_2 (110)/perovskite ($FA_{0.83}Cs_{0.17}Pb(I_{0.83}Br_{0.17})_3$) **B:** stretched exponential fitting of glass/perovskite ($FA_{0.83}Cs_{0.17}Pb(I_{0.83}Br_{0.17})_3$) and **C:** stretched exponential fitting of TiO_2 (110)/perovskite ($FA_{0.83}Cs_{0.17}Pb(I_{0.83}Br_{0.17})_3$). The excitation of the samples was at 467 nm and the decay traces were monitored at 750 nm.

Table S7: Fitting parameters used for the various fitting methods in the evaluation of the TRPL decay traces.

	**Sample**	**Method**	**A_1**	**τ_1 (ns)**	**A_2**	**τ_2 (ns)**	**β**	**A_3**	**τ_3 (ns)**	**τ_{eff} (ns)**
Exponential fitting	Glass/perovskite	Bi-exponential	-	-	0.74	22.1	-	0.26	84.2	28.0
	TiO_2/perovskite	Bi-exponential	-	-	0.82	11.2	-	0.18	49.0	13.5
		Tri-exponential	0.43	3.4	0.49	17.6	-	0.08	64.9	-
Stretched exponential fitting	Glass/perovskite	Bi-exponential			0.88	23.9	0.88	0.12	117.7	-
	TiO_2/perovskite	Add one more exponential component	0.69	11.6	0.29	23.9	0.88	0.02	117.8	-

TA data evaluation

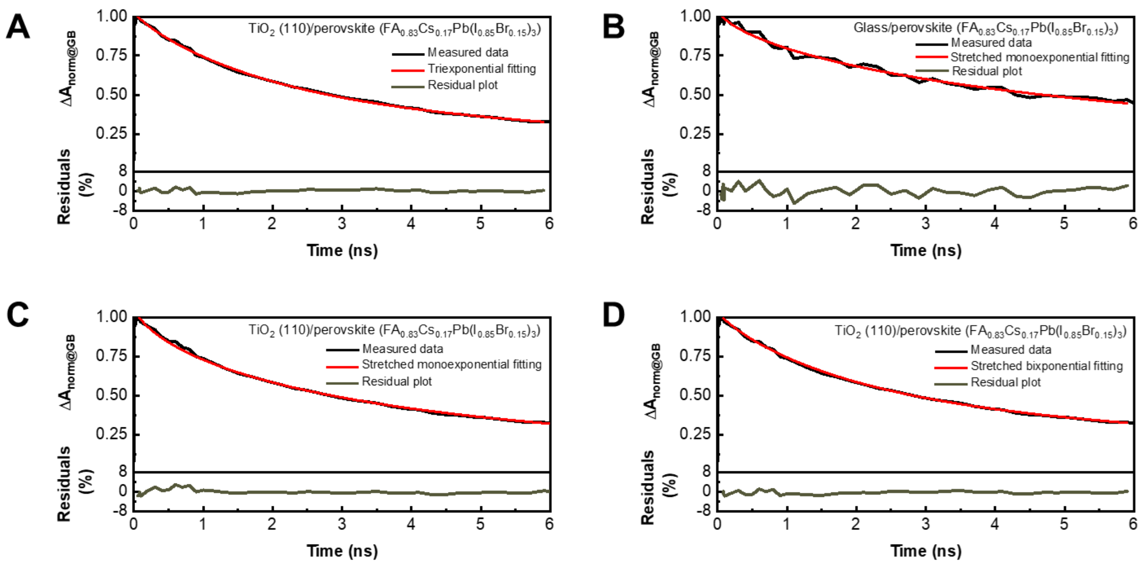

Figure S2: Representative TA decay curves graphed together with the fitting curve and the residual of **A:** triexponential fitting of TiO_2 (110)/perovskite ($FA_{0.83}Cs_{0.17}Pb(I_{0.83}Br_{0.17})_3$) **B:** stretched mono-exponential fitting of glass/perovskite ($FA_{0.83}Cs_{0.17}Pb(I_{0.83}Br_{0.17})_3$) **C:** stretched mono-exponential fitting of TiO_2 (110)/perovskite ($FA_{0.83}Cs_{0.17}Pb(I_{0.83}Br_{0.17})_3$) and **D:** stretched biexponential fitting of TiO_2 (110)/perovskite ($FA_{0.83}Cs_{0.17}Pb(I_{0.83}Br_{0.17})_3$). The measurements were performed following a 600 nm laser pulse excitation with pump fluences

of 5.7 µJ cm^{-2}. The decay profiles were extracted from the transient absorption spectra at 725 nm.

Table S8: Fitting parameters used for the various fitting methods in the evaluation of the TA decay traces.

	Sample	Method	A_1	τ_1 (ns)	A_2	τ_2 (ns)	A_3	τ_3 (ns)	β	τ_{eff} (ns)
Exponential fitting	Glass/ perovskite	Bi-exponential	-	-	0.26	1.1	0.74	10.8	-	7.5
	TiO_2/ perovskite	Bi-exponential	-	-	0.27	0.9	0.73	6.4	-	4.9
		Tri-exponential	0.03	0.2	0.50	1.8	0.47	11.9	-	-
Stretched exponential fitting	Glass/ perovskite	Mono-exponential	-	-	-	-	1.00	6.7	0.67	-
	TiO_2/ perovskite	Mono-exponential	-	-	-	-	1.00	3.6	0.67	-
	TiO_2/ perovskite	Bi-exponential	-	-	0.3	1.9	0.67	6.7	0.67	-

References

(1) Makuta, S.; Liu, M.; Endo, M.; Nishimura, H.; Wakamiya, A.; Tachibana, Y. Photo-Excitation Intensity Dependent Electron and Hole Injections from Lead Iodide Perovskite to Nanocrystalline TiO_2 and Spiro-OMeTAD. *Chem. Comm.* **2016**, *52* (4), 673–676.

(2) Du, B.; Wei, Q.; Cai, Y.; Liu, T.; Wu, B.; Li, Y.; Chen, Y.; Xia, Y.; Xing, G.; Huang, W. Crystal Face Dependent Charge Carrier Extraction in TiO_2/Perovskite Heterojunctions. *Nano Energy* **2020**, *67*, 104227.

(3) Zhong, D.; Cai, B.; Wang, X.; Yang, Z.; Xing, Y.; Miao, S.; Zhang, W.-H.; Li, C. Synthesis of Oriented TiO_2 Nanocones with Fast Charge Transfer for Perovskite Solar Cells. *Nano Energy* **2015**, *11*, 409–418.

(4) Kim, B. J.; Kim, M.; Lee, D. G.; Lee, G.; Bang, G. J.; Jeon, J. B.; Choi, M.; Jung, H. S. Interface Design of Hybrid Electron Extraction Layer for Relieving Hysteresis and Retarding Charge Recombination in Perovskite Solar Cells. *Adv. Mater. Interfaces* **2018**, *5* (23), 1800993.

(5) Li, Y.; Zhao, Y.; Chen, Q.; Yang, Y. (Michael); Liu, Y.; Hong, Z.; Liu, Z.; Hsieh, Y.-T.; Meng, L.; Li, Y.; Yang, Y. Multifunctional Fullerene Derivative for Interface Engineering in Perovskite Solar Cells. *J. Am. Chem. Soc.* **2015**, *137* (49), 15540–15547.

(6) Xing, G.; Wu, B.; Chen, S.; Chua, J.; Yantara, N.; Mhaisalkar, S.; Mathews, N.; Sum, T. C. Interfacial Electron Transfer Barrier at Compact $TiO_2/CH_3NH_3PbI_3$ Heterojunction. *Small* **2015**, *11* (29), 3606–3613.

(7) Zhu, Z.; Ma, J.; Wang, Z.; Mu, C.; Fan, Z.; Du, L.; Bai, Y.; Fan, L.; Yan, H.; Phillips, D. L.; Yang, S. Efficiency Enhancement of Perovskite Solar Cells through Fast Electron Extraction: The Role of Graphene Quantum Dots. *J. Am. Chem. Soc.* **2014**, *136* (10), 3760–3763.

(8) Piatkowski, P.; Cohen, B.; Javier Ramos, F.; Di Nunzio, M.; Nazeeruddin, M. K.; Grätzel, M.; Ahmad, S.; Douhal, A. Direct Monitoring of Ultrafast Electron and Hole Dynamics in Perovskite Solar Cells. *Phys. Chem. Chem. Phys.* **2015**, *17* (22), 14674–14684.

(9) Wang, L.; McCleese, C.; Kovalsky, A.; Zhao, Y.; Burda, C. Femtosecond Time-Resolved Transient Absorption Spectroscopy of $CH_3NH_3PbI_3$ Perovskite Films: Evidence for Passivation Effect of PbI $_2$. *J. Am. Chem. Soc.* **2014**, *136* (35), 12205–12208.

(10) Dursun, I.; Maity, P.; Yin, J.; Turedi, B.; Zhumekenov, A. A.; Lee, K. J.; Mohammed, O. F.; Bakr, O. M. Why Are Hot Holes Easier to Extract than Hot Electrons from Methylammonium Lead Iodide Perovskite? *Adv. Energy Mater.* **2019**, *9* (22), 1900084.

(11) Shen, Q.; Ogomi, Y.; Chang, J.; Tsukamoto, S.; Kukihara, K.; Oshima, T.; Osada, N.; Yoshino, K.; Katayama, K.; Toyoda, T.; Hayase, S. Charge Transfer and Recombination at the Metal Oxide/$CH_3NH_3PbClI_2$/Spiro-OMeTAD Interfaces: Uncovering the Detailed Mechanism behind High Efficiency Solar Cells. *Phys. Chem. Chem. Phys.* **2014**, *16* (37), 19984–19992.

(12) Grancini, G.; Viola, D.; Lee, Y.; Saliba, M.; Paek, S.; Cho, K. T.; Orlandi, S.; Cavazzini, M.; Fungo, F.; Hossain, M. I.; Belaidi, A.; Tabet, N.; Pozzi, G.; Cerullo, G.; Nazeeruddin, M. K. Femtosecond Charge-Injection Dynamics at Hybrid Perovskite Interfaces. *ChemPhysChem* **2017**, *18* (17), 2381–2389.

(13) Pydzińska, K.; Karolczak, J.; Kosta, I.; Tena-Zaera, R.; Todinova, A.; Idígoras, J.; Anta, J. A.; Ziółek, M. Determination of Interfacial Charge-Transfer Rate Constants in Perovskite Solar Cells. *ChemSusChem* **2016**, *9* (13), 1647–1659.

(14) Kim, J.; Godin, R.; Dimitrov, S. D.; Du, T.; Bryant, D.; McLachlan, M. A.; Durrant, J. R. Excitation Density Dependent Photoluminescence Quenching and Charge Transfer Efficiencies in Hybrid Perovskite/Organic Semiconductor Bilayers. *Adv. Energy Mater.* **2018**, *8* (35), 1802474.

(15) Xu, W.; Du, T.; Sachs, M.; Macdonald, T. J.; Min, G.; Mohan, L.; Stewart, K.; Lin, C.-T.; Wu, J.; Pacalaj, R.; Haque, S. A.; McLachlan, M. A.; Durrant, J. R. Asymmetric Charge Carrier Transfer and Transport in Planar Lead Halide Perovskite Solar Cells. *Cell Rep. Phys. Sci.* **2022**, *3* (5), 100890.

(16) Xing, G.; Mathews, N.; Sun, S.; Lim, S. S.; Lam, Y. M.; Grätzel, M.; Mhaisalkar, S.; Sum, T. C. Long-Range Balanced Electron- and Hole-Transport Lengths in Organic-Inorganic $CH_3NH_3PbI_3$. *Science* **2013**, *342* (6156), 344–347.

(17) Zhou, M.; Sarmiento, J. S.; Fei, C.; Wang, H. Charge Transfer and Diffusion at the Perovskite/PCBM Interface Probed by Transient Absorption and Reflection. *J. Phys. Chem. C* **2019**, *123* (36), 22095–22103.

(18) Pydzińska-Białek, K.; Szeremeta, J.; Wojciechowski, K.; Ziółek, M. Insights into the Femtosecond to Nanosecond Charge Carrier Kinetics in Perovskite Materials for Solar Cells. *J. Phys. Chem. C* **2019**, *123* (1), 110–119.

(19) Horn, J.; Minda, I.; Schwoerer, H.; Schlettwein, D. Direct Observation of Charge Injection From $CH_3NH_3PbI_{3-x}Cl_x$ to Organic Semiconductors Monitored With Sub-Ps Transient Absorption Spectroscopy. *Phys. Status Solidi B* **2019**, *256* (3), 1800265.

(20) Zheng, F.; Wen, X.; Bu, T.; Chen, S.; Yang, J.; Chen, W.; Huang, F.; Cheng, Y.; Jia, B. Slow Response of Carrier Dynamics in Perovskite Interface upon Illumination. *ACS Appl. Mater. Interfaces* **2018**, *10* (37), 31452–31461.

(21) Chen, W.; Pham, N. D.; Wang, H.; Jia, B.; Wen, X. Spectroscopic Insight into Efficient and Stable Hole Transfer at the Perovskite/Spiro-OMeTAD Interface with Alternative Additives. *ACS Appl. Mater. Interfaces* **2021**, *13* (4), 5752–5761.

(22) Zhang, H.; Liu, M.; Yang, W.; Judin, L.; Hukka, T. I.; Priimagi, A.; Deng, Z.; Vivo, P. Thionation Enhances the Performance of Polymeric Dopant-Free Hole-Transporting Materials for Perovskite Solar Cells. *Adv. Mater. Interfaces* **2019**, *6* (18), 1901036.
